# A comprehensive set of transcript sequences of the heavy metal hyperaccumulator *Noccaea caerulescens*

**DOI:** 10.3389/fpls.2014.00261

**Published:** 2014-06-20

**Authors:** Ya-Fen Lin, Edouard I. Severing, Bas te Lintel Hekkert, Elio Schijlen, Mark G. M. Aarts

**Affiliations:** ^1^Laboratory of Genetics, Wageningen UniversityWageningen, Netherlands; ^2^Laboratory of Bioinformatics, Wageningen UniversityWageningen, Netherlands; ^3^Business Unit Bioscience, Plant Research International, Wageningen University and Research CentresWageningen, Netherlands

**Keywords:** metal hyperaccumulation, metal hypertolerance, phytoremediation, zinc, cadmium, gene expression, Brassicaceae

## Abstract

*Noccaea caerulescens* is an extremophile plant species belonging to the Brassicaceae family. It has adapted to grow on soils containing high, normally toxic, concentrations of metals such as nickel, zinc, and cadmium. Next to being extremely tolerant to these metals, it is one of the few species known to hyperaccumulate these metals to extremely high concentrations in their aboveground biomass. In order to provide additional molecular resources for this model metal hyperaccumulator species to study and understand the mechanism of adaptation to heavy metal exposure, we aimed to provide a comprehensive database of transcript sequences for *N. caerulescens*. In this study, 23,830 transcript sequences (isotigs) with an average length of 1025 bp were determined for roots, shoots and inflorescences of *N. caerulescens* accession “Ganges” by Roche GS-FLEX 454 pyrosequencing. These isotigs were grouped into 20,378 isogroups, representing potential genes. This is a large expansion of the existing *N. caerulescens* transcriptome set consisting of 3705 unigenes. When translated and compared to a Brassicaceae proteome set, 22,232 (93.2%) of the *N. caerulescens* isotigs (corresponding to 19,191 isogroups) had a significant match and could be annotated accordingly. Of the remaining sequences, 98 isotigs resembled non-plant sequences and 1386 had no significant similarity to any sequence in the GenBank database. Among the annotated set there were many isotigs with similarity to metal homeostasis genes or genes for glucosinolate biosynthesis. Only for transcripts similar to *Metallothionein3* (*MT3*), clear evidence for an additional copy was found. This comprehensive set of transcripts is expected to further contribute to the discovery of mechanisms used by *N. caerulescens* to adapt to heavy metal exposure.

## Introduction

*Noccaea caerulescens* (J. & C. Presl) F. K. Mey., formerly named *Thlaspi caerulescens*, is an outstanding model plant species to study heavy metal hyperaccumulation (Assunção et al., 2003; Peer et al., 2003; Milner and Kochian, 2008). It is one of the few plant species of which genotypes are known that are adapted to grow on soil containing high levels of zinc (Zn), cadmium (Cd), nickel (Ni), and/or lead (Pb) (Mohtadi et al., 2012). Not only are these genotypes extremely tolerant to the different heavy metals they are exposed to, but *N. caerulescens* can also hyperaccumulate these metals to high concentrations in shoots. Accumulations of Zn to 30,000 μg g^−1^, Cd to 2700 μg g^−1^, and Ni to 4000 μg g^−1^ levels are reported on a shoot dry weight (dw) base. These are two orders of magnitude higher than other, non-accumulating, species generally accumulate (Reeves and Brooks, 1983; McGrath et al., 1993; Brown et al., 1995; Lombi et al., 2000). Next to *N. caerulescens, Arabidopsis halleri* is developed as model metal hyperaccumulator (Meyer and Verbruggen, 2012). This species is also hypertolerant to Zn and Cd, and a strong Zn hyperaccumulator, but less of a Cd hyperaccumulator and not known to be adapted to Ni. Unlike *N. caerulescens* it is self-incompatible, which complicates genetic analysis, but it is much closer to the general plant model species *Arabidopsis thaliana*, with which is shares more sequence synteny. Adaptation to heavy metals seems to be more commonly occurring in the Brassicaceae family than in other families, with other *Noccaea* species known to hyperaccumulate Ni and Zn, and one, *N. praecox*, also hyperaccumulating Cd, as recently reviewed by Koch and German (2013). Other genera with metal hyperaccumulating species are *Alyssum* and *Strepthanthus* (both mostly Ni adapted). Thus, it is fortunate that the first plant species for which genomic data became available, *A. thaliana*, belongs to the Brassicaceae family, as this triggered further interest in generating genome sequence information from several members of this family (http://www.brassica.info/resource/sequencing/bmap.php). For neither *A. halleri* nor *N. caerulescens* the genome sequence has been determined, which is why most of the gene expression research on these species so far has relied on heterologous micro-array analysis using the available *A. thaliana* micro-arrays (Becher et al., 2004; Weber et al., 2004; Hammond et al., 2006; Talke et al., 2006; Van De Mortel et al., 2006, 2008). This revealed that both species seem to have evolved similar strategies for dealing with the high metal exposure, typically by modifying the expression of several genes normally involved in Zn and Fe mineral homeostasis. A striking example is the copy number expansion of the *HMA4* gene, observed in both species, which increased expression of the gene when compared to non-accumulating species (Hanikenne et al., 2008; Ó Lochlainn et al., 2011; Craciun et al., 2012).

One reason to investigate the remarkable metal adaptation properties of *N. caerulescens* is that these extremophile plants are interesting target species to develop for metal phytoextraction purposes, in which plants are used to remediate soils contaminated with toxic metals (Peer et al., 2006; Anjum, 2012). Another reason is that the rare extremophile nature of metal hyperaccumulators, to have adapted to otherwise hostile environments, makes them interesting models for plant evolutionary genomics studies (Hanikenne and Nouet, 2011). For both purposes, unraveling the evolutionary and physiological mechanisms that allowed their adaptation, at the molecular level, will be needed. The main approaches that have been followed so far in identifying genes involved in metal adaptation involve comparisons of hyperaccumulating and related non-hyperaccumulating plant species, either by using genetic crosses, or by transcriptomics or proteomics comparisons (reviewed by Krämer et al., 2007; Hassan and Aarts, 2011; Lin and Aarts, 2012). However, so far, a comprehensive set of genome sequences of a metal hyperaccumulator species is not available, which is seriously limiting the progress in molecular analysis of metal adaptation.

Molecular analysis of *N. caerulescens* has been largely performed based on its close relationship to the well-known model species *A. thaliana*, allowing the use of molecular tools and genomic databases developed for this species. Although the lineages of both species separated probably some 20 Mya (Clauss and Koch, 2006), there still is substantial genome sequence similarity between these species, estimated at 87–88% sequence identity in intergenic transcribed spacer regions and 88.5% sequence identity in transcribed regions (Peer et al., 2003; Rigola et al., 2006). This high level of conservation was sufficient to use heterologous, *A. thaliana*, micro-arrays for comparative transcriptome analyses (Hammond et al., 2006; Van De Mortel et al., 2006, 2008). The first attempt to obtain sequence information of *N. caerulescens* was performed by Rigola et al. (2006), who generated an Expressed Sequence Tag (EST) database of little over 3700 transcript sequences. This resource has been used to generate a cDNA-based micro-array, which has been used for transcript profiling of *N. caerulescens*, but due to the limited number of probes on the array, the information that could be gathered was limited (Plessl et al., 2010). Since then, the rapid development of high-throughput sequencing techniques has made whole genome and transcriptome sequencing a lot more efficient and affordable (Morozova et al., 2009). Recently, SOLiD technology has been applied for comparative root transcriptomics of three different *N. caerulescens* accessions (Halimaa et al., 2014). The 454/Roche pyrosequencing method takes advantages of long read lengths and a fast running time (Metzker, 2010; Niedringhaus et al., 2011) and is a suitable method for whole transcriptome sequencing, when the main purpose is to obtain a comprehensive set of transcript sequences of substantial length to be used as a reference database for future transcriptome profiling studies.

Here we present such a comprehensive set of *N. caerulescens* transcripts, which largely exceeds the previous dataset in the number of identified genes (Rigola et al., 2006). We identified many transcripts involved in mineral accumulation and homeostasis, which may be relevant for metal hyperaccumulation and hypertolerance. In addition we listed genes involved in the biosynthesis of glucosinolates. These are secondary metabolites conferring resistance to herbivores, especially prominent in Brassicaceae (Bones and Rossiter, 1996). This new transcript sequence information will facilitate the further analysis of the extremophile traits of *N. caerulescens* and is expected to contribute to similar studies in related metal hyperaccumulators, such as *A. halleri*, and also less studied species like the Zn/Cd hyperaccumulator *Noccaea praecox* or the Ni-hyperaccumulator *Noccaea goesingense* (Koch and German, 2013). It will also contribute to more efficient functional studies of heavy metal related genes, to establish their role in metal adaptation or for possible applications in metal phytoextraction, or genes related to synthesis of glucosinolates.

## Materials and methods

### Plant materials and RNA preparation

An inbred line of *N. caerulescens* accession “Ganges” (kindly obtained from Dr. Henk Schat, Free University, Amsterdam, The Netherlands) was used. Roots and shoots were collected separately from 5-week-old plants, grown in half-strength Hoagland solutions (Assunção et al., 2001) containing 10 μM ZnSO_4_, in a climate controlled growth chamber (set at 20/15°C day/night temperature; 70% relative humidity; 12 h day time). Inflorescences were collected from 22-week-old plants grown in soil. RNA of these three plant parts was extracted separately by using the RNeasy® Plant Mini kit (Qiagen, cat. no. 74904) with on-column RNase-Free DNase set digestions (Qiagen, cat. no. 79254). The RNA concentrations were quantified using the highly selective Qubit™ RNA BR Assay kit (Invitrogen™, cat. no. Q10210) with a Qubit® 2.0 Fluorometer.

### cDNA library construction

Similar amounts of RNA from roots, shoots, and flowers were pooled and used for preparation of a normalized and random-primed cDNA library for Roche/454 sequencing (Vertis Biotechnologie AG). From the total RNA sample, poly(A)^+^ RNA was isolated, and used for cDNA synthesis. First strand cDNA synthesis was primed using random hexamer primers. To the 5′ and 3′ ends of the cDNA, Roche/454 adapters A and B, as provided by the manufacturer, were ligated and cDNA was finally amplified using 12 PCR cycles and proofreading DNA polymerase. Normalization was performed by denaturation and re-association of cDNA. Re-associated double-stranded cDNA was separated from remaining (normalized) single-stranded cDNA (ss-cDNA) over a hydroxylapatite column. After separation, ss-DNA was PCR-amplified using six PCR cycles. Finally cDNA in the size range of 500–850 bp was eluted from a preparative agarose gel. The final normalized cDNA library contains double stranded fragments of between 500 and 850 bp, consisting of the following sequence structure: 5′-454-Adapter A (CCA-TCT-CAT-CCC-TGC-GTG-TCT-CCG-ACT-CAG), 5′- barcode (CACACG), 5′ adapter (GAC-CTT-GGC-TGT-CAC-TCA-GTT), cDNA insert (400–750 bp), 3′ adapter (TCG-CAG-TGA-GTG-ACA-GGC-CA), 3′-454-Adapter B (CTG-AGA-CTG-CCA-AGG-CAC-ACA-GGG-GAT-AGG).

### *De novo* sequencing and assembly of *n. caerulescens* transcript sequences

Prior to Roche/454 sequencing, cDNA library molecules were clonally amplified using a “two copies per bead” ratio for one Large Volume Emulsion PCR, following the manufacturer's protocol (Roche, Genome Sequencer FLX Titanium Series). Two million DNA carrying beads (from a 23% enrichment) were loaded on half a picotiter plate equivalent, divided over two regions. Sequencing was done on a 454 GS FLX Titanium instrument using XLR70 chemistry and 200 flow cycles. The raw reads were assembled after adaptor trimming using Newbler (454 life Sciences Corporation) version v 2.6.

### Sequence annotation and characterization

The predicted proteomes and genome assemblies of *A. thaliana, Arabidopsis lyrata, Brassica rapa, Capsella rubella*, and *Thellungiella halophila*, recently suggested to be named *Eutrema halophila* (Koch and German, 2013), were downloaded from the phytozome repository version 9.1 (Goodstein et al., 2012). The protein function annotation of *A. thaliana* version TAIR10.0 was obtained from the TAIR website (www.arabidopsis.org). The gene ontology (GO) annotation for *A. thaliana* was downloaded from the GO webpage (www.geneontology.org). The protein annotations (including GO-terms) of the four other species were downloaded from the phytozome repository. Pathway and Enzyme Code annotations for *A. thaliana* and *B. rapa* were downloaded from the PlantCyc database (www.plantcyc.org) version 8.0. The annotated GO terms were summarized using the plant GOSlim Set of the GOSlim viewer at the AgBase website (http://www.agbase.msstate.edu/cgi-bin/tools/goslimviewer_select.pl) (McCarthy et al., 2006).

The predicted Brassicaceae proteins were clustered into groups of orthologous proteins by first performing all 25 possible pairwise similarity searches between the five predicted proteomes using BlastP version 2.26 (Altschul et al., 1990). Pairwise orthologs were determined for all 10 possible species-pairs using Inparanoid version 4.1 (Remm et al., 2001). Finally, multi-species-ortholog clusters were built by feeding the Inparanoid output files to the multiparanoid Perl-script (Alexeyenko et al., 2006). Whenever possible, the annotation of orthologous protein clusters was inherited from *A. thaliana* or *B. rapa* members (in that order). Clusters without *A. thaliana* or *B. rapa* members remained un-annotated.

The transcriptome of *N. caerulescens* was first searched against the predicted proteomes using BlastX with an e-value cut-off of 10^−5^. Sequences for which the best hit was a member of an annotated cluster inherited the annotation from that cluster. The remaining sequences inherited their annotation from their best hit. Sequences without a significant match against the predicted Brassica proteomes were searched (BlastX with e-value cut-off 10^−5^) against the non-redundant protein database (NR) downloaded from the NCBI ftp-site (ftp://ftp.ncbi.nlm.nih.gov/). Proteins without BlastX hits were searched against the genomes of the Brassica species using BLAT (Kent, 2002) version 35. BLAT hits were filtered by requiring 90% of the *N. caerulescens* sequence to be aligned with an average identity of at least 85%. Finally sequences were searched against the NR nucleotide database, downloaded from the NCBI ftp-site (BlastN with e-value cut-off 10^−5^). Blast hits were only accepted if the corresponding HSPs had ≥85% sequence identity and covered ≥90% of the query isotigs.

Protein alignments were constructed using ClustalW2 (Larkin et al., 2007) and regions of low alignment quality were removed by hand using JalView (Waterhouse et al., 2009). Maximum likelihood trees were constructed using PhyML (the following command line parameters were used: -c 4 -m LG -o lr -v e -a e -f e -b 100) (Guindon et al., 2010). In brief, the trees were constructed using the LG amino acid substitution model (Le and Gascuel, 2008) with four relative substitution rate categories. The proportion of invariable sites and equilibrium amino-acid frequencies were estimated from the data. The alpha parameter was estimated by maximizing the likelihood of the phylogeny. Branching patterns were validated using bootstrap-analysis; 100 bootstrap-samples were generated for the ML-trees and 1000 for the NJ trees. Trees were displayed using the ETE2 python package (Huerta-Cepas et al., 2010) and dendroscope (Huson and Scornavacca, 2012). To investigate the presence of gene duplications of metal homeostasis related genes, sequence sets were created for each gene family implicated in metal homeostasis by performing blast searches against the proteomes of the five Brassicaceae reference species (with e-value cut-off 10^−5^). The exonerate program was used to rapidly compare transcript sequences to genome sequences (Slater and Birney, 2005). Neighbor-joining trees were subsequently constructed for all alignments using ClustalW2. This Transcriptome Shotgun Assembly project has been deposited at DDBJ/EMBL/GenBank under the accession GASZ00000000. The version described in this paper is the first version, GASZ01000000. Raw sequence data have been deposited in the NCBI short read archive under the accession SRX456668.

## Results

### *De novo* sequencing and assembly of *n. caerulescens* isotigs

To maximize the transcript diversity, a pool of RNA from roots, shoots, and inflorescences of *N. caerulescens* was made. A cDNA library synthesized from this RNA pool was used for sequencing. In total 834,911 raw reads with an average length of 401 nucleotides (nt) were obtained and subsequently assembled using Newbler. During the assembly process, two initial contigs can be joined together into an isotig when raw reads are found that map to the ends of both contigs. The resulting transcriptome assembly (Table 1) consisted of 26,785 contigs the vast majority of which were further assembled into a set of 23,836 putative transcripts (isotigs) with an average sequence length of 1025 base pairs (bp). Isotigs sharing at least one contig are grouped into isogroups. Isotigs are likely to be transcript isoforms from the same gene (Isogroup). The isotigs in this study were grouped into 20,378 isogroups. In total 2326 isogroups (11.4%) consisted of more than one isotig. The distributions of the isotigs sizes and the number of isotigs per isogroup are provided in Supplemental Figure S1.

**Table 1:** **Summary of the *N. caerulescens* transcriptome analysis**.

Transcript assembly	Number
Total reads	834,911
Total bases	3^*^10^8^
Percentage aligned reads	86.24
No. assembled reads	638,451
**ISOTIGS**	
No. isotigs	23,836
No. bases	24,420,501
Average isotig size	1025
N50 isotig size	1185
Largest isotig size	6573
**ISOGROUPS**	
No. isogroups	20,378
Average no. isotigs/isogroup	1.2
Largest isotig count	77
No. isogroups with one isotig	18,052
Q40 Plus bases	97.3 %

The Newbler software was used to assemble raw sequence reads into isotigs (representing putative transcripts) and isogroups (representing putative genes). N50 is the length for which the collection of all isotigs of that length or longer contains at least half of the total of the lengths of the isotigs. Quality value 40 (Q40) means the error rate is below 1 in 10,000 nucleotides.

### Annotation and functional characterization of *n. caerulescens* genes

**Figure 1:**
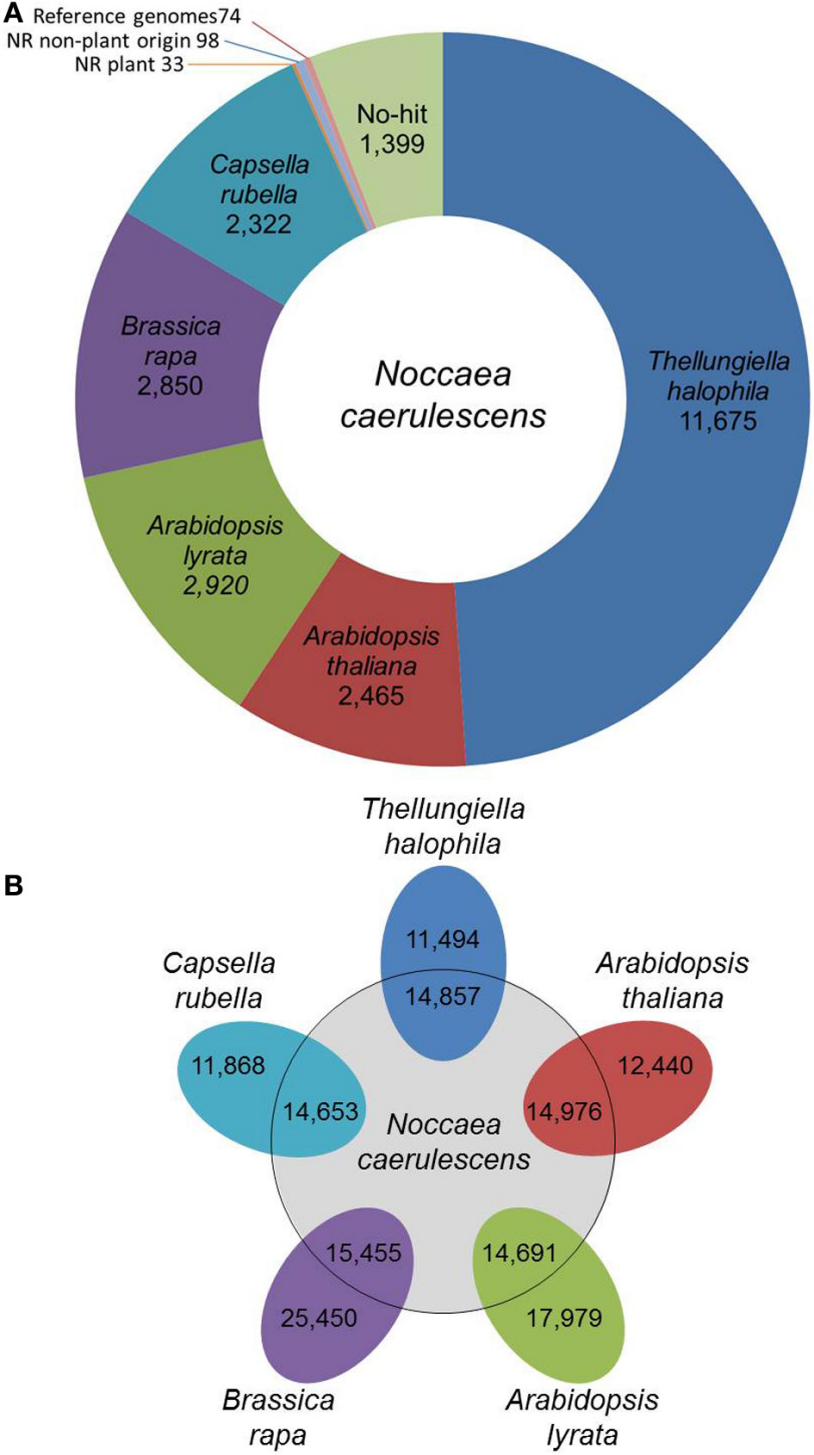
**Classification of *Noccaea caerulescens* isotigs according to best-hits. (A)** The number of best-hits of *N. caerulescens* isotigs to the Brassicaceae proteome (comprising those of *Thellungiella (Eutrema) halophila, Arabidopsis thaliana, Arabidopsis lyrata, Brassica rapa*, and *Capsella rubella*) are listed. The 1604 isotigs without any significant hit to Brassicaceae proteome were further compared to the NCBI NR protein database, identifying best-hit similarities to 33 proteins of plant origin and 98 of non-plant origin. The non-hit isotigs were further matched to the available genome sequences of the listed Brassicaceae reference species, identifying another 74 best-hit similarities. Finally, a set of 1399 isotigs remained for which no protein similarity could be found. **(B)** Venn diagram showing the number of genes in each of the five reference species that correspond to the best-hit in that species for each *N. caerulescens* isotig sequence.

*N. caerulescens* is a member of the Brassicaceae family, which is why we used the annotation of five other Brassicaceae species of which full genome sequence is available (*A. thaliana, A. lyrata, B. rapa, C. rubella*, and *T. halophila*) as a reference for annotation. The predicted proteomes of these five reference species were first clustered in 24,172 groups of orthologous proteins, which we called the Brassicaceae proteome (see Materials and Methods). In total 23,691 of these clusters were annotated following the *A. thaliana* or *B. rapa* annotation. We then compared the *N. caerulescens* isotig DNA sequences against the Brassicaceae proteome and listed the best match (Summarized in Figure 1A; full list in Supplemental Table S1). A total of 22,232 (93.2%) *N. caerulescens* isotigs (19191 isogroups) had a significant match with the Brassicaceae proteome and we annotated these sequences according to their best match. We also determined the best match for each *N. caerulescens* sequence in each of the five reference species (Figure 1B). Based on this analysis, we estimate that between 40 and 60% of all *N. caerulescens* protein encoding genes are represented in this transcriptome set.

Even though five closely related species were used as reference, still 1604 isotigs were found without any significant hit in the Brassicaceae proteome data set. These isotigs were therefore compared with the NCBI NR protein database. The search revealed that, although not detected in our initial search, 33 isotigs did have a significant match to a plant protein, including 16 isotigs that strongly resembled an *A. thaliana* protein and seven that resembled proteins from other Brassicaceae (Supplemental Table S2). Thirteen of the 33 isotigs corresponded to (retro)transposons and nearly all of the remaining ones encoded for hypothetical or unknown proteins. Next to these, an additional 98 isotigs showed only significant similarity to non-plant sequences (Supplemental Table S3). Based on the high similarity to genes of organisms often found to be associated with plants (fungi, bacteria, viruses, etc.), these are most likely reflecting such associations, rather than actual *N. caerulescens* genes. The remaining sequences with no hit when compared to the NR protein database, were compared against the genome sequences of the five Brassicaceae reference species. This revealed an additional 74 sequences with significant DNA matches to one or more of the reference genomes. Still, there are 1399 isotigs that had no hit to Brassica proteins and proteins in the NR database (Supplemental Table S4). When these were compared to the NR DNA database, an additional 13 isotigs were found to be similar to DNA entries in the database. Only two of these were plant genes. Five isotigs showed similarity to *A. thaliana* genomic sequences, one to plant mitochondrial DNA and the remaining five appeared to correspond to microbial sequences.

The list of isotigs with best-hit matches to the Brassicaceae proteome (Supplemental Table S1) has been used to obtain an estimate of transcript length coverage of the *N. caerulescens* isotigs (Supplemental Figure S2). This shows that around half of the transcripts cover over 80% of the protein sequence of their Brassicaceae best-hit match. In addition, we compared the full list of *N. caerulescens* isotigs to the previously obtained set of *N. caerulescens* ESTs (Rigola et al., 2006) using BlastN (≥95% identity and ≥60% coverage of shorter sequences). This retrieved 3773 of the 4289 sequences (88%). Sequence similarities between both datasets were generally around 98%, suggesting SNP frequencies of around 2 per 100 bp.

### Gene ontology (GO) annotation of *n. caerulescens* isogroups

Subsequently, we used the *N. caerulescens* isogroup sequences to categorize putative genes according to GO, by assigning them to three categories of GO terms: Biological Process, Cellular Compartment, and Molecular Function (see Materials and Methods) (Supplemental Table S5). The annotated GO terms were further classified with the plant set of the GO Slim Viewer (McCarthy et al., 2006) (summarized in Supplemental Figure S3, full list in Supplemental Table S6).

Within Biological Process, two major categories are cellular process (25.1%) and metabolic process (19.3%), followed by developmental process (12.3%), and response to stress/stimulus (10.4%). Genes involved in cellular ion homeostasis (involving “cations” and “metal,” but also specifically zinc, copper, calcium, iron, manganese, potassium, and phosphate) (0.1%), are likely to be crucial for the regulation of the heavy metal balance in *N. caerulescens* (Supplemental Figure S3), in addition to genes involved in transport (2.7%), especially ion transport (0.5%), and signal transduction (1.6%). Within Cellular Compartment, the largest category is cytoplasmic component (50.7%), followed by membrane apparatus (12%), and then intracellular components (10.7%), extracellular components (2.1%), and cell wall (0.9%). Genes encoding proteins localized to the plasma membrane (5%) and the vacuolar membrane (0.6%) will comprise metal transporter genes that are expected to be involved in metal uptake and sequestration (Supplemental Figure S3). Within Molecular Function, the largest category is binding activity (37.1%) comprising ion binding (4%) as an important sub-category for metal hyperaccumulation traits. Furthermore, the genes categorized for transporter activity (8.1%) are expected to play an important role in metal uptake and metal transportation. To see if this is different from other species, we also compared *N. caerulescens* and *A. thaliana* regarding the GO terms with the highest percentages of *N. caerulescens* isogroup counts (Supplemental Figure S3). This made clear that there are only minor differences between both species.

A total of 3051 isotigs (2610 isogroups) could be assigned to 352 PlantCyc pathways (Supplemental Table S7). The 26 most represented pathways (around 20 or more isogroups) are shown in Supplemental Figure S4. The top six pathways are triacylglycerol degradation, homogalacturonan degradation, glycolysis II (from fructose-6P), tRNA charging, aerobic respiration (alternative oxidase pathway), and betanidin degradation, which have more than 50 isogroups found in the pathway.

### Transcripts related to the response to heavy metal exposure

The metal hyperaccumulation and hypertolerance properties of *N. caerulescens* partly rely on a number of genes known in other species, mainly *A. thaliana*, to be involved in mineral homeostasis. A list of such genes, including 87 isogroups, is shown in Table 1. The list includes metal transporter genes belonging to the ZRT/IRT-like Protein (*ZIP*) gene family (9 isogroups), the Natural Resistance-Associated Macrophage Protein (*NRAMP*) family (8 isogroups), the Heavy Metal ATPase (*HMA*) family (9 isogroups), the Metal Tolerance Protein (*MTP*) family (7 isogroups), and the Calcium exchanger (*CAX*) family (9 isogroups). In addition, we listed genes belonging to the Plant Cadmium Resistance (PCR) gene family (2 isogroups), the Pleiotropic Drug Resistance protein (*PDR*) family (14 isogroups), and Plant Defensin (*PDF*) family (8 isogroups). Furthermore, transcripts related to metal chelator and metal chelator transporter functions were listed, such as genes of the Nicotianamine Synthase (*NAS*) family (4 isogroups), the Phytochelatin Synthase (*PCS*) family, (2 isogroups), the Metallothionein (*MT*) family (4 isogroups), the Yellow Stripe Like protein (*YSL*) family, (6 isogroups), the Zinc Induced Facilitator (*ZIF*) family (5 isogroups), and the Multidrug Resistance-associated Protein/ ATP-binding cassette transporter ABCC type (*MRP/ABCC*) family (26 isogroups). *Nota bene* we did not include the large families of genes encoding heavy metal-associated isoprenylated plant proteins (HIPP) or heavy metal-associated plant proteins (HPP), of which some members are recently suggested to be relevant for response to Cd (De Abreu-Neto et al., 2013).

For two larger gene families, the ZIP and MTP families, we performed a phylogenetic analysis (**Figures 3A,B**) to confirm that the Blast analysis (Table 2) indeed identified the most likely *A. thaliana* orthologs. Of the ZIP family, no transcripts were found for orthologs of *AtIRT2, AtZIP3, AtZIP5, AtZIP7, AtZIP8*, and *AtZIP12*. For the MTP family, we did not find *N. caerulescens* orthologs of the *AtMTP2, AtMTP3, AtMTP4, AtMTP9*, and *AtMTP10* genes. Next we searched for *N. caerulescens* specific gene duplications for all gene classes in Table 2. To this end we created for each class multiple sequence alignments with sequences from the five reference species that were similar to the *N. caerulescens* isogroup sequences (see Materials and Methods). Neighbor-joining trees were constructed from these alignments and inspected manually for putative *N. caerulescens* specific duplications. Only in the tree of the MT3 class we identified a putative *N. caerulescens* specific duplication. In order to obtain additional support for a recent gene duplication we identified the *MT3* orthologs of the five Brassicaceae reference species and constructed a phylogenetic tree of them (**Figure 3C**). This confirmed that all inspected genomes had only one copy of the *MT3* gene.

**Table 2:** ***Noccaea caerulescens* isogroups corresponding to *A. thaliana* genes involved in mineral homeostasis and heavy metal stress response**.

**Gene name**	**Isogroup no**.	**Longest isotig length**	**Best hit**	***E*-value**	**Annotation source**	**Annotation source description**
*bZIP19*	isogroup05302	1413	Carubv10005538m|PACid:20894480	1.49E-152	AT4G35040	Basic-leucine zipper (bZIP) transcription factor
*IRT1*	isogroup16405	559	AT4G19690.2|PACid:19648297	1.31E-110	AT4G19690	Iron-regulated transporter 1
*IRT3*	isogroup00440	1428	Thhalv10023475m|PACid:20201432	0	AT1G60960	Iron regulated transporter 3
*ZIP1*	isogroup00441	1341	Carubv10015186m|PACid:20899838	2.19E-110	AT3G12750	Zinc transporter 1 precursor
*ZIP2*	isogroup07896	1085	Thhalv10013963m|PACid:20204020	0	AT5G59520	ZRT/IRT-like protein 2
*ZIP4*	isogroup06106	1285	AT1G10970.1|PACid:19650740	0	AT1G10970	Zinc transporter 4 precursor
*ZIP6*	isogroup06071	1293	Thhalv10016885m|PACid:20179820	5.49E-180	AT2G30080	ZIP metal ion transporter family
*ZIP9*	isogroup09981	918	Thhalv10025584m|PACid:20196090	1.39E-169	AT4G33020	ZIP metal ion transporter family
*ZIP10*	isogroup06264	1263	Thhalv10007930m|PACid:20188086	1.39E-165	AT1G31260	Zinc transporter 10 precursor
*ZIP11*	isogroup09047	988	AT1G55910.1|PACid:19649515	0	AT1G55910	Zinc transporter 11 precursor
*MTP1/ZAT*	isogroup00207	2104	AT2G46800.2|PACid:19641115	2.92E-85	AT2G46800	Zinc transporter of Arabidopsis thaliana
*MTP5*	isogroup01278	1466	Thhalv10020889m|PACid:20182053	0	AT3G12100	Cation efflux family protein
*MTP6*	isogroup10156	907	Thhalv10001431m|PACid:20189077	9.05E-167	AT2G47830	Cation efflux family protein
*MTP7*	isogroup03594	1841	Thhalv10011469m|PACid:20184771	0	AT1G51610	Cation efflux family protein
*MTP8*	isogroup05294	1416	Thhalv10006008m|PACid:20190151	0	AT3G58060	Cation efflux family protein
*MTP11*	isogroup07586	1117	497437|PACid:16043531	0	AT2G39450	Cation efflux family protein
*MTP12*	isogroup17157	526	Thhalv10027647m|PACid:20189919	6.90E-11	AT2G04620	Cation efflux family protein
*NRAMP1*	isogroup00017	2604	Thhalv10018389m|PACid:20191829	0	AT1G80830	Natural resistance-associated macrophage protein 1
*NRAMP2*	isogroup18133	488	Thhalv10011381m|PACid:20184824	6.26E-43	AT1G47240	NRAMP metal ion transporter 2
*NRAMP3*	isogroup03577	1845	Bra000573|PACid:22711711	0	AT2G23150	Natural resistance-associated macrophage protein 3
*NRAMP4*	isogroup06857	1197	Thhalv10004033m|PACid:20199053	0	AT5G67330	Natural resistance associated macrophage protein 4
*NRAMP5*	isogroup13240	720	Thhalv10026903m|PACid:20193643	1.53E-159	AT4G18790	NRAMP metal ion transporter family protein
*EIN2-NRAMP*	isogroup05270	1417	AT5G03280.1|PACid:19671290	0	AT5G03280	NRAMP metal ion transporter family protein
	isogroup05627	1367	Thhalv10012456m|PACid:20203745	0	AT5G03280	NRAMP metal ion transporter family protein
	isogroup11803	801	Thhalv10012456m|PACid:20203745	7.09E-37	AT5G03280	NRAMP metal ion transporter family protein
*HMA1*	isogroup17702	504	Carubv10004142m|PACid:20894021	1.03E-48	AT4G37270	Heavy metal atpase 1
	isogroup03139	2048	490893|PACid:16045505	0	AT4G37270	Heavy metal atpase 1
*HMA2*	isogroup08856	1001	Thhalv10024341m|PACid:20194930	3.36E-139	AT4G30110	Heavy metal atpase 2
	isogroup10109	910	Bra024107|PACid:22710111	4.02E-103	Bra024107	Heavy metal atpase 2
	isogroup02580^*^	2745	857597|PACid:16066225	0	Bra011165	Heavy metal atpase 2
*HMA4*	isogroup00021	4417	Carubv10012822m|PACid:20897962	0	AT2G19110	Heavy metal atpase 4
*HMA6/PAA1*	isogroup07162	1157	AT4G33520.1|PACid:19645606	1.41E-96	AT4G33520	P-type ATP-ase 1
*HMA7/RAN1*	isogroup11167	839	Thhalv10000758m|PACid:20202842	3.25E-166	AT5G44790	Copper-exporting ATPase (RAN1)
*HMA8/PAA2*	isogroup02963	2165	Carubv10000184m|PACid:20908511	0	AT5G21930	P-type ATPase of Arabidopsis 2
*YSL1*	isogroup02761	2382	Bra013764|PACid:22706007	0	Bra013764	YELLOW STRIPE like 1
*YSL3*	isogroup01006	2083	Thhalv10012886m|PACid:20203959	0	AT5G53550	YELLOW STRIPE like 3
*YSL5*	isogroup03348	1934	Thhalv10020160m|PACid:20182600	0	AT3G17650	YELLOW STRIPE like 5
*YSL6*	isogroup00783	2244	Thhalv10003758m|PACid:20198225	0	AT3G27020	YELLOW STRIPE like 6
*YSL7*	isogroup17698	497	Thhalv10018216m|PACid:20192307	1.52E-84	AT1G65730	YELLOW STRIPE like 7
	isogroup12528	757	Thhalv10018216m|PACid:20192307	1.23E-138	AT1G65730	YELLOW STRIPE like 7
*ZIF1*	isogroup05885	1322	Thhalv10013388m|PACid:20203275	8.58E-175	AT5G13740	Zinc induced facilitator 1
*ZIFL1*	isogroup13593	697	Bra008805|PACid:22694599	3.84E-111	AT5G13750	Zinc induced facilitator-like 1
	isogroup14641	646	Thhalv10013417m|PACid:20206568	1.30E-136	AT5G13750	Zinc induced facilitator-like 1
*ZIFL2*	isogroup02224	478	Bra021053|PACid:22721993	1.81E-60	AT3G43790	Zinc induced facilitator-like 2
	isogroup08493	1027	Bra021053|PACid:22721993	3.28E-139	AT3G43790	Zinc induced facilitator-like 2
*NAS1*	isogroup15107	622	Thhalv10014123m|PACid:20206622	3.97E-119	AT5G04950	Nicotianamine synthase 1
*NAS2*	isogroup06206	1273	Thhalv10013812m|PACid:20203757	0	AT5G56080	Nicotianamine synthase 2
*NAS3*	isogroup09071	987	Thhalv10008206m|PACid:20187253	0	AT1G09240	Nicotianamine synthase 3
*NAS4*	isogroup06921	1184	Thhalv10024166m|PACid:20201706	0	AT1G56430	Nicotianamine synthase 4
*MRP1/ABCC1*	isogroup04168	1656	473263|PACid:16034489	1.04E-12	AT1G30400	Multidrug resistance-associated protein 1
	isotig01105^a^	1975	AT1G30400.2|PACid:19655137	3.18E-06	AT1G30400	Multidrug resistance-associated protein 1
	isogroup11349	828	Thhalv10006546m|PACid:20187496	6.64E-96	AT1G30400	Multidrug resistance-associated protein 1
*MRP2/ABCC2*	isotig01102^a^	4514	Thhalv10016133m|PACid:20181262	6.34E-06	AT2G34660	Multidrug resistance-associated protein 2
	isogroup13185	724	482430|PACid:16055424	3.02E-83	AT2G34660	Multidrug resistance-associated protein 2
*MRP3/ABCC3*	isogroup17760	504	Thhalv10019893m|PACid:20181977	4.35E-23	AT3G13080	Multidrug resistance-associated protein 3
	isogroup02973	2159	Thhalv10019893m|PACid:20181977	0	AT3G13080	Multidrug resistance-associated protein 3
	isogroup08374	1033	AT3G13080.1|PACid:19658374	3.01E-12	AT3G13080	Multidrug resistance-associated protein 3
*MRP4/ABCC4*	isotig00322^b^	5401	Bra018722|PACid:22695420	0	AT2G47800	Multidrug resistance-associated protein 4
	isotig00323^b^	5294	Bra018722|PACid:22695420	0	AT2G47800	Multidrug resistance-associated protein 4
	isotig00324^b^	5063	Bra018722|PACid:22695420	0	AT2G47800	Multidrug resistance-associated protein 4
	isotig00326^b^	4956	Bra018722|PACid:22695420	0	AT2G47800	Multidrug resistance-associated protein 4
*MRP5/ABCC5*	isogroup02478	3126	Carubv10008087m|PACid:20891448	0	AT1G04120	Multidrug resistance-associated protein 5
	isogroup10208	901	Thhalv10006549m|PACid:20186680	0	AT1G04120	Multidrug resistance-associated protein 5
	isogroup14209	665	Thhalv10006549m|PACid:20186680	3.34E-38	AT1G04120	Multidrug resistance-associated protein 5
*MRP6/ABCC8*	isogroup15243	615	Thhalv10019895m|PACid:20181972	3.67E-53	AT3G21250	Multidrug resistance-associated protein 6
	isogroup07630	1109	Thhalv10019895m|PACid:20181972	3.29E-07	AT3G21250	Multidrug resistance-associated protein 6
*MRP7/ABCC7*	isogroup20180	277	Bra034706|PACid:22686665	9.37E-37	AT3G13100	Multidrug resistance-associated protein 7
	isogroup12703	742	Thhalv10019894m|PACid:20183109	1.81E-11	AT3G13100	Multidrug resistance-associated protein 7
	isogroup14335	661	Bra034706|PACid:22686665	3.50E-136	AT3G13100	Multidrug resistance-associated protein 7
*MRP9/ABCC9*	isogroup04767	1517	Bra003402|PACid:22702084	6.74E-14	AT3G60160	Multidrug resistance-associated protein 9
	isogroup09719	937	486482|PACid:16051413	7.50E-07	AT3G60160	Multidrug resistance-associated protein 9
*MRP10/ABCC14*	isotig00325^b^	5040	Thhalv10005741m|PACid:20190810	0	AT3G62700	Multidrug resistance-associated protein 10
	isotig00327^b^	4933	Thhalv10005741m|PACid:20190810	0	AT3G62700	Multidrug resistance-associated protein 10
*MRP14/ABCC10*	isogroup15495	603	AT3G59140.1|PACid:19663037	6.18E-119	AT3G59140	Multidrug resistance-associated protein 14
	isogroup18407	480	486382|PACid:16034938	5.72E-91	AT3G59140	Multidrug resistance-associated protein 14
*PCR2*	isogroup15392	608	AT1G14870.1|PACid:19649994	1.51E-88	AT1G14870	Plant Cadmium Resistance 2
	isogroup15934	581	Bra026796|PACid:22713063	1.43E-111	AT1G14870	Plant Cadmium Resistance 2
*PDR1*	isogroup19618	414	Thhalv10019897m|PACid:20182879	1.37E-66	AT3G16340	Pleiotropic drug resistance 1
	isogroup02600	2692	Thhalv10019897m|PACid:20182879	8.02E-38	AT3G16340	Pleiotropic drug resistance 1
*PDR3*	isogroup14595	649	AT2G29940.1|PACid:19639436	6.90E-12	AT2G29940	Pleiotropic drug resistance 3
*PDR4*	isogroup02662	2535	Thhalv10001880m|PACid:20200369	4.23E-33	AT2G26910	Pleiotropic drug resistance 4
	isogroup03404	1906	Thhalv10001880m|PACid:20200369	3.89E-17	AT2G26910	Pleiotropic drug resistance 4
*PDR5*	isogroup08926	999	870083|PACid:16064851	3.50E-09	AT2G37280	Pleiotropic drug resistance 5
	isogroup11286	836	Thhalv10016141m|PACid:20179582	4.60E-08	AT2G37280	Pleiotropic drug resistance 5
*PDR6*	isogroup07295	1148	AT2G36380.1|PACid:19640612	6.09E-16	AT2G36380	Pleiotropic drug resistance 6
	isogroup11080	843	Thhalv10016140m|PACid:20180787	2.61E-09	AT2G36380	Pleiotropic drug resistance 6
	isogroup13734	691	Thhalv10016140m|PACid:20180787	2.58E-10	AT2G36380	Pleiotropic drug resistance 6
*PDR7*	isogroup01130	1897	471713|PACid:16040487	4.48E-14	AT1G15210	Pleiotropic drug resistance 7
	isogroup12430	764	Carubv10012050m|PACid:20892263	2.05E-11	AT1G15210	Pleiotropic drug resistance 7
	isogroup14785	638	Bra026157|PACid:22695455	4.48E-126	Bra026157	Pleiotropic drug resistance 7
*PDR9/PIS1*	isogroup14778	637	Bra003137|PACid:22699106	1.69E-99	AT3G53480	Pleiotropic drug resistance 9
*CAX1*	isogroup01205	1707	Thhalv10016611m|PACid:20180411	0	AT2G38170	Cation exchanger 1
*CAX2*	isotig04417^c^	925	Thhalv10020744m|PACid:20182084	3.06E-164	AT3G13320	Cation exchanger 2
	isotig01832^d^	974	Thhalv10020744m|PACid:20182084	9.22E-94	AT3G13320	Cation exchanger 2
*CAX4*	isogroup16996	532	Bra009640|PACid:22694292	5.20E-82	AT5G01490	Cation exchanger 4
*CAX5*	isotig04418^c^	682	Thhalv10011487m|PACid:20184135	1.59E-79	AT1G55730	Cation exchanger 5
	isotig01833^d^	876	Carubv10009154m|PACid:20889697	3.20E-93	AT1G55730	Cation exchanger 5
*CAX7*	isogroup07282	1146	488652|PACid:16035533	3.74E-179	AT5G17860	Calcium exchanger 7
	isogroup15879	583	488652|PACid:16035533	4.17E-79	AT5G17860	Calcium exchanger 7
*CAX9*	isogroup06161	1278	897667|PACid:16066029	0	AT3G14070	Cation exchanger 9
*PCS1*	isotig03039^e^	1670	Thhalv10003194m|PACid:20208031	0	AT5G44070	Phytochelatin synthase 1 (PCS1)
*PCS2*	isotig03040^e^	1095	Thhalv10007644m|PACid:20187384	0	AT1G03980	Phytochelatin synthase 2
*MT2a*	isogroup01808	910	Bra029765|PACid:22685892	6.28E-24	Bra029765	Metallothionein 2A
	isogroup17171	526	Thhalv10015167m|PACid:20205299	1.01E-16	AT3G09390	Metallothionein 2A
*MT3*	isogroup17490	513	Thhalv10021853m|PACid:20182985	4.64E-24	AT3G15353	Metallothionein 3
	isogroup19215	444	Thhalv10021853m|PACid:20182985	6.02E-27	AT3G15353	Metallothionein 3
*PDF1.2*	isogroup16374	560	Thhalv10019396m|PACid:20192145	6.62E-42	AT5G44420	Plant defensin 1.2
	isogroup19505	425	Bra015811|PACid:22701509	5.12E-40	AT5G44420	Plant defensin 1.2

Gene class abbreviation are: bZIP, basic-leucine zipper; ZIP/IRT, ZRT/IRT-like Protein; MTP, Metal Tolerance Protein; NRAMP, Natural Resistance-Associated Macrophage Protein; HMA, Heavy Metal ATPase; YSL, Yellow Stripe Like protein; ZIF, Zinc Induced Facilitator; NAS, Nicotianamine Synthase; MRP/ABCC, Multidrug Resistance-associated Protein; PCR, Plant Cadmium Resistance; PDR, Pleiotropic Drug Resistance Protein; CAX, Calcium exchanger; PCS, Phytochelatin Synthase; MT, Metallothionein; PDF, Plant Defensin.

a These isotigs were both grouped into isogroup00190.

b These isotigs were all grouped into isogroup00027.

c These isotigs were both grouped into isogroup01669.

d These isotigs were both grouped into isogroup00399.

e These isotigs were both grouped into isogroup00980.

* This isogroup was found to be most similar to AtHMA3.

### Glucosinolate biosynthesis transcripts in *n. caerulescens*

Next to the mineral homeostasis genes, we also examined the occurrence of transcripts representing genes in the glucosinolate biosynthesis pathway. This pathway is generally prominent in Brassicaceae as it generates glucosinolates that upon interaction with myrosinase enzymes release toxic isothiocyanates, thiocyanates, or nitrils, which display strong anti-feeding properties against herbivores (Bones and Rossiter, 1996). Most of the genes in this pathway and their roles have been identified (Sønderby et al., 2010b; Wang et al., 2011). Important genes are a series of *MYB* and *MYC* genes encoding the transcription factors controlling aliphatic and indolic glucosinolate biosynthesis (Gigolashvili et al., 2007; Sønderby et al., 2010a; Schweizer et al., 2013), next to genes involved in core structure formation, side-chain elongation, secondary modification and co-substrate pathways (as summarized by Sønderby et al., 2010b) and more recently identified genes involved in secondary modification of indolic glucosinolates and genes encoding glucosinolate transporters (Pfalz et al., 2011; Nour-Eldin et al., 2012). *N. caerulescens* plants originating from metalliferous soils were found to contain lower levels of glucosinolates than plants originating from non-metalliferous soils (Noret et al., 2007), which is why we were interested to see if the glucosinolate genes known from *A. thaliana* were also present in the *N. caerulescens* transcriptome. Supplemental Table S8 shows the list of genes involved in the glucosinolate biosynthesis pathway, which we classified based on their presumed function as transcription factor genes, genes involved in side-chain elongation, core structure formation and secondary modification or genes of a co-substrate pathway (according to Wang et al., 2011). Of the 61 genes in the list, 40 were found to be expressed in the *N. caerulescens* transcriptome.

## Discussion

*N. caerulescens* is one of the two heavy metal hyperaccumulator plant model species, together with *A. halleri*, that are studied in detail to understand their adaptation to growing on soil containing extremely high concentrations of Zn and Cd (Verbruggen et al., 2013). As part of this adaptation, they not only tolerate exposure to normally lethal concentrations of both metals, but also hyperaccumulate them in their shoots, probably as protection against herbivory and microbial infection (Hoerger et al., 2013). *N. caerulescens* is the only species that also hyperaccumulates Ni and Pb.

The development of new and cost-effective gene expression analysis methods such as RNA-Seq prompted us to generate a comprehensive reference transcriptome dataset for *N. caerulescens* that will facilitate and support such gene expression analyses, without having to rely on heterologous comparisons. This data set replaces a previous data set we made, comprising only 4289 transcript sequences, representing 3709 unigenes, of accession “La Calamine” (Rigola et al., 2006). We now assembled 23,836 isotigs, further condensed into 20,378 isogroups of accession “Ganges,” which is a better Cd-hyperaccumulator than “La Calamine.” When assuming that isogroups will best represent genes, we have expanded the available transcriptome sequence information, in terms of sequenced genes, by almost 5.5-fold.

The use of isotigs, rather than contigs for listing transcripts, will account for small sequence differences between contigs corresponding to the same transcript. Such can be caused by sequence errors, allelic differences, or differences between recently duplicated gene copies. The accession “Ganges” was used for transcriptome sequencing. This accession had been inbred for at least seven generations, and as *N. caerulescens* is a self-compatible, readily self-fertilizing species, we expect that only few sequence differences between contigs will be due to allelic variation and that most of the differences between contigs and isotigs will be due to sequencing errors. The difference between isotigs and isogroups is mainly accounted for by differential or alternative splicing or generation of alternative transcripts from the same gene. The 454 sequence technology that was used generates relatively large reads and facilitates easier assembly compared to that of the much shorter reads created by the Illumina sequencing technology. However, despite the long reads, for about 10–15% of the isogroups more than one isotig was assembled, reflecting the occurrence of alternative transcripts (Supplemental Table S1).

When comparing the *N. caerulescens* transcriptome to that of other diploid Brassicaceae species for which transcriptome sequence is available, such as *Thlaspi arvense* (Dorn et al., 2013) or *Thellungiella salsuginea* (*Eutrema salsugineum*) (Lee et al., 2013), the *N. caerulescens* transcriptome set is relatively small. The draft transcriptome of *T. arvense* consists of 33,873 contigs, representing 25,232 Brassicaceae genes, and that of *T. salsuginea* comprises 42,810 unigenes, corresponding to 24,457 *A. thaliana* peptides. The 20,378 *N. caerulescens* isogroups corresponded to 19,191 protein matches in the Brassicaceae reference proteome set. The main reason for these differences will be the representation of more tissues or organs and conditions in the other sequenced libraries. For *N. caerulescens*, only roots, leaves and flowers of plants grown under normal Zn supply conditions were sampled, while material from more organs or conditions were sampled for the other two species. The different sequencing approaches may also account for the differences. *T. arvense* was sequenced using Illumina technology, which generates many more, but shorter, reads, while for *T. salsuginea* both a normalized and non-normalized library were sequenced, using 454 technology, and in the final assembly, previously generated EST sequences present in the NCBI GenBank database were included.

*T. salsuginea* is predicted to contain 26,521 protein encoding genes (Yang et al., 2013), of which 18,970 were found to be represented in the 42,810 unigene set (Lee et al., 2013). Similar gene numbers are also found for *Leavenworthia alabamica* (30,343 genes) and *Sisymbrium irio* (28,917 genes) (Haudry et al., 2013). These three species have a genome size comparable to the genome size of *N. caerulescens*, which is expected to be 310–330 Mb as based on 2C DNA content (Peer et al., 2003). Assuming that *N. caerulescens* has around 26,000–30,000 genes, this means around 60% of its transcriptome is covered in the current dataset.

*N. caerulescens* belongs to the Coluteocarpeae tribe of the Brassicaceae that consists mainly of *Noccaea* species, which, when tested, are all found to be metal hyperaccumulators (Koch and German, 2013). Some of the other species in this tribe, e.g., *Raparia bulbosa* and *Thlaspiceras oxyceras*, are also known to accumulate metals. This tribe belongs to the expanded lineage II of the Brassicaceae, to which also the *Brassica* and *Thellungiella* (*Eutrema*) genera belong. This fits well with the observation that most *N. caerulescens* transcript sequences find their best BLASTX hit with *T. halophila* proteins (Supplemental Table S1). Only 98 sequences appeared to be originating from non-plant organisms, potentially corresponding to organisms living in association with the *N. caerulescens* plants we used to generate the cDNA libraries. Considering that for the *T. arvense* transcriptome set, 424 out of 33,873 contigs showed similarity to fungal genes, the contribution of such genes to the *N. caerulescens* dataset is modest. Upon comparing the *N. caerulescens* isotigs to existing sequence information available in the NCBI GenBank, 1386 isotigs (5.8%) were found to have no significant similarity to any other sequence in database (Supplemental Table S4). Potentially these could be genes unique to *N. caerulescens*; however, they may also represent miRNA precursors that are much less conserved and hard to detect using Blast analysis. Of course it is possible that these sequences represent genomic DNA sequences (not very likely considering the use of DNAses in the cDNA library construction), or transcripts of plant-associated organisms that have not been sequenced yet. In the previous *N. caerulescens* transcriptome dataset we generated, we found 8% of the unigenes to show no similarity to any other entry in the NCBI database (Rigola et al., 2006). Comparison of the “no-hit” sequences to the *N. caerulescens* whole genome sequence, when available, will be needed to clarify if these are from *N. caerulescens*.

**Figure 2:**
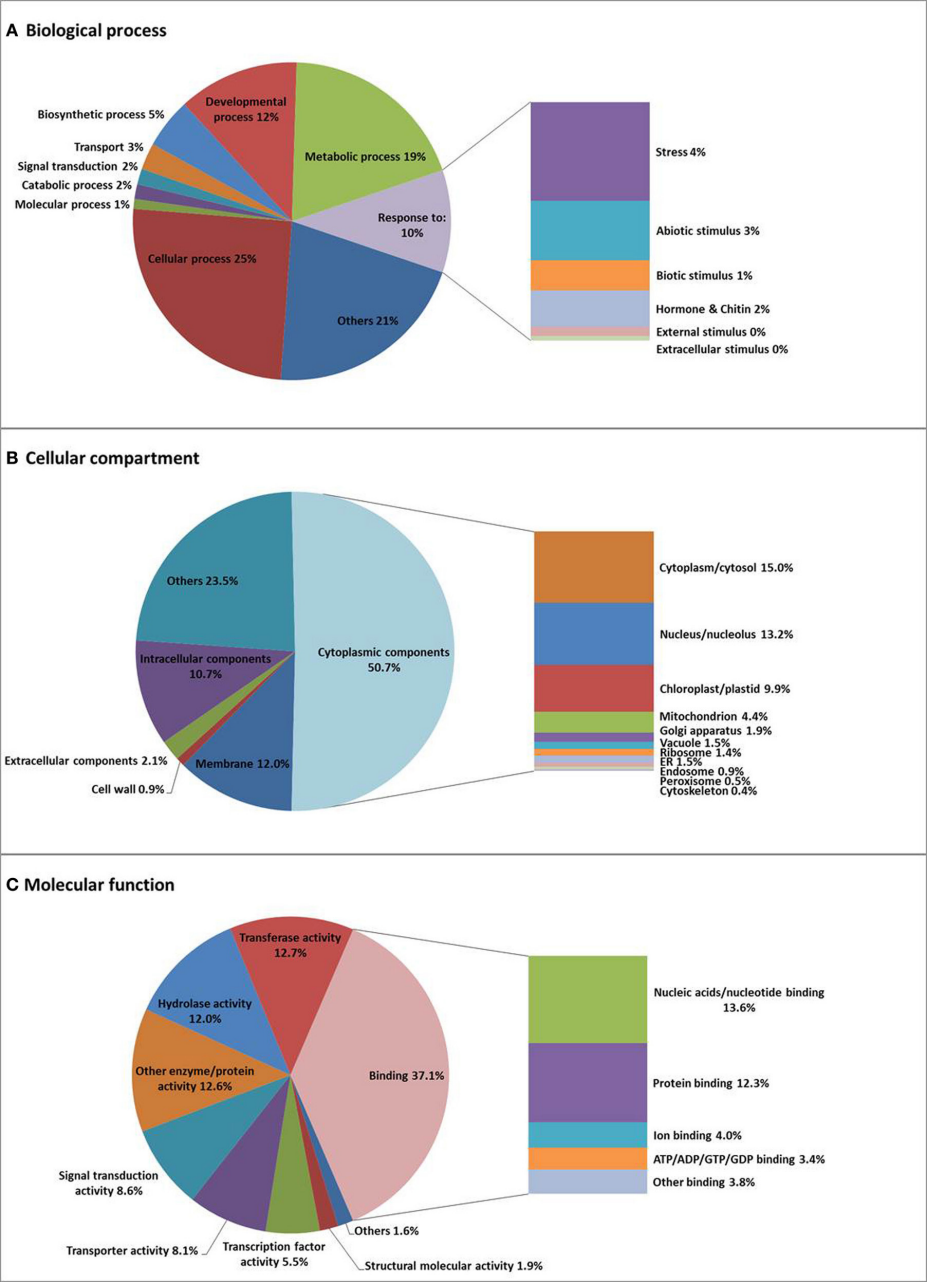
**Gene Ontology (GO) classification of *N. caerulescens* isogroups**. *N. caerulescens* isogroups were classified into three major functional GO groups: Biological Process **(A)**, Cellular Component **(B)**, and Molecular Function **(C)** and subsequently sub-classified as indicated.

Isogroups rather than isotigs were used for GO annotation and analysis to avoid overrepresentation of certain GO terms due to alternative transcripts generating many isotigs for the same gene. Sometimes the isogrouping appears to be too strict, which has forced transcripts from different paralogs into one isogroup, as was found for some of the mineral homeostasis related genes (Table 2). The GO analysis (Figure 2, Supplemental Figure S3 and Supplemental Tables S5 and S6) showed that *N. caerulescens* isogroups were largely GO-annotated in comparable GO-class representations as for *A. thaliana*.

**Figure 3:**
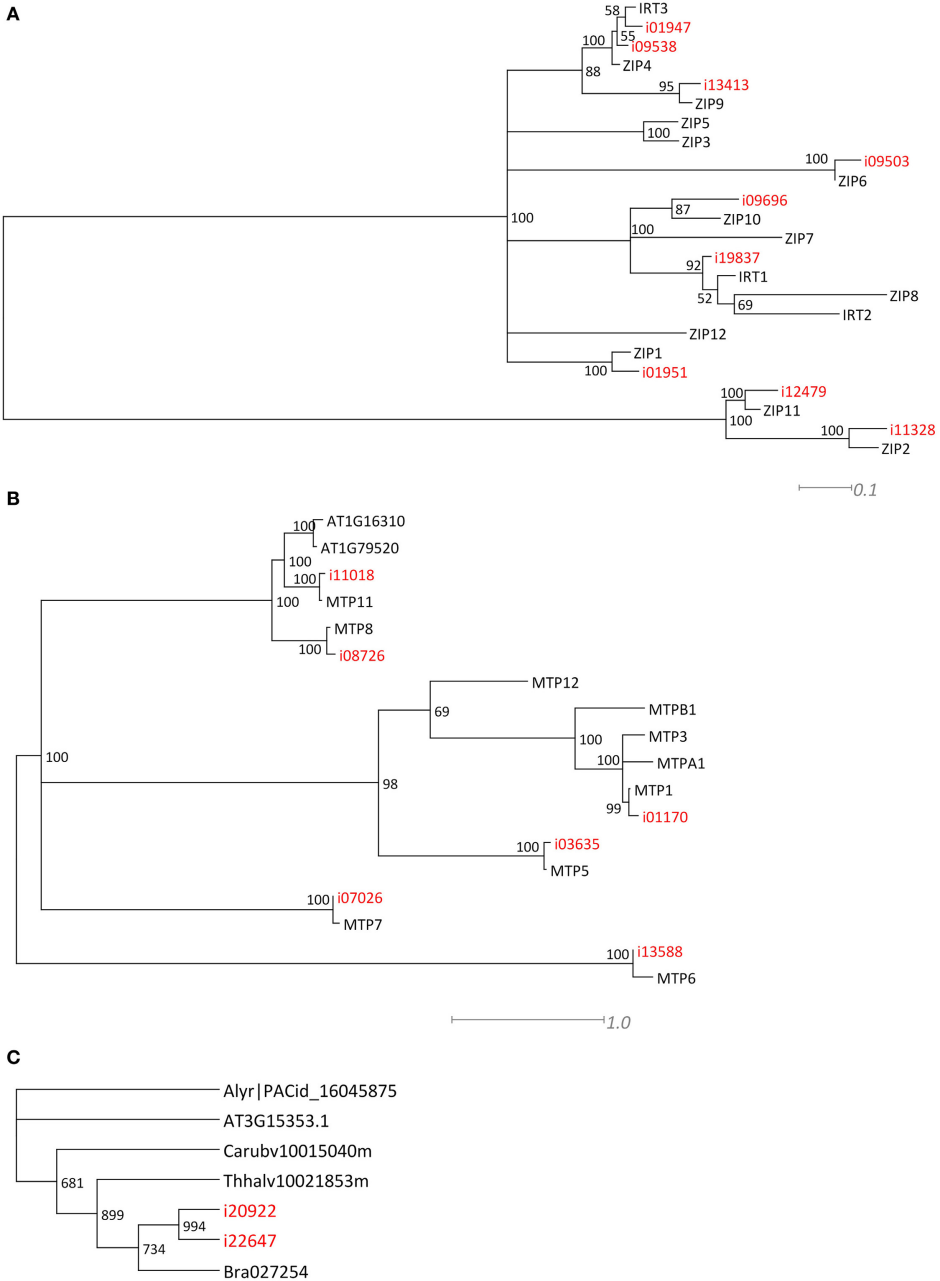
**Phylogenetic comparison of *ZIP, MTP*, and *MT* related *N. caerulescens* isotigs**. Maximum Likelihood trees, showing the longest isotigs (indicated in red) belonging to different isogroups of the **Z**RT/**I**RT-like **P**rotein (*ZIP*) **(A)** and **M**etal **T**olerance **P**rotein (*MTP*) gene families **(B)** as identified in Table 2, and compared at amino acid sequence level to *A. thaliana* genes to indicate the most likely orthologs. When all proteins listed in Table 2 were manually examined for *N. caerulescens* specific gene duplications, this appeared to be the case only for **M**etallo**t**hionein 3 (*MT3*). The Neighbor Joining tree is shown in **(C)**, displaying the comparison of nucleotide sequences of *MT3* isotigs to most similar genes of the previously used five Brassicaceae reference gene sets, indicates that *N. caerulescens* expresses an additional gene copy. Note that all branches in the Maximum Likelihood and Neighbor Joining trees with 50% or less bootstrap support were collapsed.

Many mineral homeostasis genes were identified among the *N. caerulescens* isotigs (Table 2). Although we identified genes for all relevant gene families, not all genes for each family were found to have *N. caerulescens* counterparts, representing their potential orthologs. For instance, for the *ZIP* gene family of plasma membrane metal importers, no *N. caerulescens* transcript was found for *IRT2* and *ZIP3, 5, 7, 8*, and *12*. Transcripts for these genes have been found by Halimaa et al. (2014), but except for the *ZIP5* ortholog, these are expressed at low levels in GA roots. Not finding a potential ortholog for *ZIP5* is remarkable, since transcripts for these genes were also found previously (Rigola et al., 2006) and at least the ortholog of *ZIP5* was expressed in roots and shoots (Wu et al., 2009). Also for the *MTP* family of vacuolar metal importers we did not detect transcripts similar to all *A. thaliana MTP* genes, but most of them have been found by Halimaa et al. (2014). Phylogenetic analysis of the *ZIP* and *MTP* isotigs showed that those were classified correctly (Figure 3). For the *HMA* family of plasma membrane metal exporter genes, we initially did not find a potential ortholog of the *NcHMA3* gene (Ueno et al., 2011). However, upon re-examining the isogroups showing similarity to HMA proteins, isogroup02580, which was found to be very similar to a *B. rapa* protein annotated as HMA2, was actually highly similar (~90% DNA identity) to *A. halleri* and *A. thaliana HMA3* genes.

Only for the *MT3* gene we could identify an additional copy in *N. caerulescens* compared to the single copies found in related species (Figure 3C). This gene has been implicated in Cu homeostasis, and expression in a southern France accession (“St. Felix,” which is close to the origin of “Ganges”) is much higher than in the two other tested accessions and appears to be constitutive, rather than induced by Cu (Roosens et al., 2004). Such could well be the consequence of an additional copy, which would mean the additional copy is accession, rather than species specific, and is worth further investigations. Not finding copy number expansion for other genes is remarkable, as for several of the other genes listed in Table 2, such as *HMA3* and *HMA4*, multiple copies have already been reported in other accessions, including “Ganges” (Ó Lochlainn et al., 2011; Ueno et al., 2011; Craciun et al., 2012; Iqbal et al., 2013). However, for *HMA4*, for which cDNA sequences of “Ganges” are available, the different cDNA copies are very similar in sequence (Iqbal et al., 2013), only differing in a few bp and three InDels, which will not be distinguished from sequence differences due to sequencing errors by the assembly software we used. This is likely to be the case for more recently duplicated gene copies. Detailed analysis of copy number variation will be much less ambiguous upon availability of a whole genome sequence, where non-encoding sequence can be taken into account to distinguish duplicated copies.

When examining genes involved in glucosinolate biosynthesis, we identified isotigs corresponding to all transcription factor genes involved in aliphatic glucosinolates, but not for two of the *MYB* genes involved in indolic glucosinolates (*MYB34* and *MYB122*) (Supplemental Table S8). In contrast, hardly any of the aliphatic side-chain elongation genes as well as the two CYP79F genes involved in aliphatic core structure formation was represented in the *N. caerulescens* isogroup list. Of course, not finding them in the isotig list does not mean these genes are not expressed, but it is remarkable that expression of several of the structural genes involved in aliphatic glucosinolate biosynthesis is so low that apparently these genes are more likely to missed by 454 sequencing than the other genes. Low expression of glucosinolate genes would be in line with a previous report that especially metallicolous accessions from the south of France, where also “Ganges” is originating from, are low in total glucosinolate levels (Noret et al., 2007).

The main reason for studying *N. caerulescens* is to learn more on its extraordinary capacities to tolerate exposure to high concentrations of heavy metals and accumulate these to extremely high levels in the leaves. The transcriptome sequence as such will not tell us that much on which genes will be relevant for these traits, but it can be used as an excellent reference for RNA-Seq studies to determine gene expression in organs of different accessions, exposed to a range of metal concentrations. There are many different *N. caerulescens* populations in Europe, which can differ substantially in their ability to tolerate and accumulate metals, as well as differ in their metal preferences. Many populations grow on non-metallicolous soils, which are not enriched in metals. At those sites, they are likely to accumulate mainly Zn. When exposed to Zn, Ni, and Cd they can hyperaccumulate them, but as they are generally not metal tolerant, plants will rapidly die upon exposure. Other natural populations grow on (ultramafic) serpentine outcrops, which are generally rich in metals, but not Zn. At those sites, they often hyperaccumulate Ni, and when exposed to Zn or Cd, will also hyperaccumulate these metals in their shoot. However, they are often only tolerant to Ni and will suffer or die from exposure to Cd. Finally, there are several local populations spread over different sites in Europe that have been heavily contaminated by Zn and Cd, and often also Pb (Mohtadi et al., 2012). These calamine populations are Zn and often Cd hyperaccumulating and are also tolerant to these metals. When exposed to Ni, they will also hyperaccumulate it, but they are less tolerant to it than populations from serpentine sites (Assunção et al., 2003). Especially populations found on metal contaminated sites in the Cevennes region, around the town of Ganges in the south of France, are particularly good at hyperaccumulating Cd. Most of the phenotypic differences related to metal tolerance and accumulation between populations are due to genetic differences, often reflected in differences in gene expression (Van De Mortel et al., 2008). With the *N. caerulescens* transcriptome dataset we generated, it will be much easier to study such differences. Also, when the whole genome sequence of *N. caerulescens* will be determined, the transcriptome data can be used to annotate predicted genes and delineate potential transcripts of such genes. This will be useful to determine the function of these genes. Finally, since the previous transcriptome dataset was obtained from accession “La Calamine,” and the new one from “Ganges,” the comparison of transcripts from the same genes revealed a SNP frequency of around 2 per 100 bp. Although the presence of sequence errors in both sets will have inflated this number, it will be straightforward to convert these differences into genetic markers that can be used in mapping quantitative trait loci in segregating populations of *N. caerulescens* (Assunção et al., 2006; Deniau et al., 2006).

### Conflict of interest statement

The authors declare that the research was conducted in the absence of any commercial or financial relationships that could be construed as a potential conflict of interest.

## References

[B1] Alexeyenko A. Tamas I. Liu G. Sonnhammer E. L. L. (2006). Automatic clustering of orthologs and inparalogs shared by multiple proteomes. Bioinformatics 22, e9–e15 10.1093/bioinformatics/btl213 16873526

[B2] Altschul S. Gish W. Miller W. Myers E. Lipman D. (1990). Basic local alignment search tool. J. Mol. Biol. 215, 403–410 2231712 10.1016/S0022-2836(05)80360-2

[B3] Anjum N. A. (2012). The Plant Family Brassicaceae Contribution Towards Phytoremediation [Online]. Dordrecht; New York: Springer 10.1007/978-94-007-3913-0

[B4] Assunção A. G. L. Da Costa Martins P. De Folter S. Vooijs R. Schat H. Aarts M. G. M. (2001). Elevated expression of metal transporter genes in three accessions of the metal hyperaccumulator *Thlaspi caerulescens*. Plant Cell Environ. 24, 217–226 10.1111/j.1365-3040.2001.00666.x

[B5] Assunção A. G. L. Pieper B. Vromans J. Lindhout P. Aarts M. G. M. Schat H. (2006). Construction of a genetic linkage map of *Thlaspi caerulescens* and quantitative trait loci analysis of zinc accumulation. New Phytol. 170, 21–32 10.1111/j.1469-8137.2005.01631.x 16539600

[B5a] Assunção A. G. L. Schat H. Aarts M. G. M. (2003). *Thlaspi caerulescens*, an attractive model species to study heavy metal hyperaccumulation in plants. New Phytol. 159, 351–360 10.1046/j.1469-8137.2003.00820.x 33873356

[B6] Becher M. Talke I. N. Krall L. Krämer U. (2004). Cross-species microarray transcript profiling reveals high constitutive expression of metal homeostasis genes in shoots of the zinc hyperaccumulator *Arabidopsis halleri*. Plant J. 37, 251–268 10.1046/j.1365-313X.2003.01959.x 14690509

[B7] Bones A. M. Rossiter J. T. (1996). The myrosinase-glucosinolate system, its organisation and biochemistry. Physiol. Plant. 97, 194–208 10.1111/j.1399-3054.1996.tb00497.x

[B8] Brown S. Angle J. Chaney R. Baker A. (1995). Zinc and cadmium uptake by hyperaccumulator *Thlaspi caerulescens* grown in nutrient solution. Soil Sci. Soc. Am. J. 59, 125–133 22276881 10.1021/es00006a022

[B9] Clauss M. J. Koch M. A. (2006). Poorly known relatives of *Arabidopsis thaliana*. Trends Plant Sci. 11, 449–459 10.1016/j.tplants.2006.07.005 16893672

[B10] Craciun A. R. Meyer C.-L. Chen J. Roosens N. De Groodt R. Hilson P. (2012). Variation in HMA4 gene copy number and expression among *Noccaea caerulescens* populations presenting different levels of Cd tolerance and accumulation. J. Exp. Bot. 63, 4179–4189 10.1093/jxb/ers104 22581842

[B11] De Abreu-Neto J. B. Turchetto-Zolet A. C. De Oliveira L. F. V. Bodanese Zanettini M. H. Margis-Pinheiro M. (2013). Heavy metal-associated isoprenylated plant protein (HIPP): characterization of a family of proteins exclusive to plants. FEBS J. 280, 1604–1616 10.1111/febs.12159 23368984

[B12] Deniau A. Pieper B. Ten Bookum W. Lindhout P. Aarts M. Schat H. (2006). QTL analysis of cadmium and zinc accumulation in the heavy metal hyperaccumulator *Thlaspi caerulescens*. Theor. Appl. Gene. 113, 907–920 10.1007/s00122-006-0350-y 16850314

[B13] Dorn K. M. Fankhauser J. D. Wyse D. L. Marks M. D. (2013). De novo assembly of the pennycress (*Thlaspi arvense*) transcriptome provides tools for the development of a winter cover crop and biodiesel feedstock. Plant J. 75, 1028–1038 10.1111/tpj.12267 23786378 PMC3824206

[B15] Gigolashvili T. Berger B. Mock H.-P. Müller C. Weisshaar B. Flügge U.-I. (2007). The transcription factor HIG1/MYB51 regulates indolic glucosinolate biosynthesis in *Arabidopsis thaliana*. Plant J. 50, 886–901 10.1111/j.1365-313X.2007.03099.x 17461791

[B16] Goodstein D. M. Shu S. Howson R. Neupane R. Hayes R. D. Fazo J. (2012). Phytozome: a comparative platform for green plant genomics. Nucleic Acids Res. 40, D1178–D1186 10.1093/nar/gkr944 22110026 PMC3245001

[B17] Guindon S. Dufayard J.-F. Lefort V. Anisimova M. Hordijk W. Gascuel O. (2010). New algorithms and methods to estimate maximum-likelihood phylogenies: assessing the performance of PhyML 3.0. Syst. Biol. 59, 307–321 10.1093/sysbio/syq010 20525638

[B18] Halimaa P. Lin Y.-F. Ahonen V. H. Blande D. Clemens S. Gyenesei A. (2014). Gene expression differences between *Noccaea caerulescens* ecotypes help to identify candidate genes for metal phytoremediation. Environ. Sci. Technol. 48, 3344–3353 10.1021/es4042995 24559272

[B19] Hammond J. P. Bowen H. C. White P. J. Mills V. Pyke K. A. Baker A. J. M. (2006). A comparison of the *Thlaspi caerulescens* and *Thlaspi arvense* shoot transcriptomes. New Phytol. 170, 239–260 10.1111/j.1469-8137.2006.01662.x 16608451

[B20] Hanikenne M. Nouet C. (2011). Metal hyperaccumulation and hypertolerance: a model for plant evolutionary genomics. Curr. Opin. Plant Biol. 14, 252–259 10.1016/j.pbi.2011.04.003 21531166

[B21] Hanikenne M. Talke I. N. Haydon M. J. Lanz C. Nolte A. Motte P. (2008). Evolution of metal hyperaccumulation required cis-regulatory changes and triplication of HMA4. Nature 453, 391–395 10.1038/nature06877 18425111

[B22] Hassan Z. Aarts M. G. M. (2011). Opportunities and feasibilities for biotechnological improvement of Zn, Cd or Ni tolerance and accumulation in plants. Environ. Exp. Bot. 72, 53–63 10.1016/j.envexpbot.2010.04.003

[B23] Haudry A. Platts A. E. Vello E. Hoen D. R. Leclercq M. Williamson R. J. (2013). An atlas of over 90,000 conserved noncoding sequences provides insight into crucifer regulatory regions. Nat. Genet. 45, 891–898 10.1038/ng.2684 23817568

[B24] Hoerger A. C. Fones H. N. Preston G. (2013). The current status of the elemental defense hypothesis in relation to pathogens. Front. Plant Sci. 4:395 10.3389/fpls.2013.00395 24137169 PMC3797420

[B25] Huerta-Cepas J. Dopazo J. Gabaldón T. (2010). ETE: a python environment for tree exploration. BMC Bioinformatics 11:24 10.1186/1471-2105-11-24 20070885 PMC2820433

[B26] Huson D. H. Scornavacca C. (2012). Dendroscope 3: an interactive tool for rooted phylogenetic trees and networks. Syst. Biol. 61, 1061–1067 10.1093/sysbio/sys062 22780991

[B27] Iqbal M. Nawaz I. Hassan Z. Hakvoort H. W. J. Bliek M. Aarts M. G. M. (2013). Expression of *HMA4* cDNAs of the zinc hyperaccumulator *Noccaea caerulescens* from endogenous *NcHMA4* promoters does not complement the zinc-deficiency phenotype of the *Arabidopsis thaliana hma2hma4* double mutant. Front. Plant Sci. 4:404 10.3389/fpls.2013.00404 24187545 PMC3807671

[B28] Kent W. J. (2002). BLAT—the blast-like alignment tool. Genome Res. 12, 656–664 10.1101/gr.229202 11932250 PMC187518

[B29] Koch M. A. German D. (2013). Taxonomy and systematics are key to biological information: Arabidopsis, Eutrema (Thellungiella), Noccaea and Schrenkiella (Brassicaceae) as examples. Front. Plant Sci. 4:267 10.3389/fpls.2013.00267 23914192 PMC3728732

[B30] Krämer U. Talke I. N. Hanikenne M. (2007). Transition metal transport. FEBS Lett. 581, 2263–2272 10.1016/j.febslet.2007.04.010 17462635

[B31] Larkin M. A. Blackshields G. Brown N. P. Chenna R. McGettigan P. A. McWilliam H. (2007). Clustal W and Clustal X version 2.0. Bioinformatics 23, 2947–2948 10.1093/bioinformatics/btm404 17846036

[B32] Le S. Q. Gascuel O. (2008). An improved general amino acid replacement matrix. Mol. Biol. Evol. 25, 1307–1320 10.1093/molbev/msn067 18367465

[B33] Lee Y. Giorgi F. Lohse M. Kvederaviciute K. Klages S. Usadel B. (2013). Transcriptome sequencing and microarray design for functional genomics in the extremophile Arabidopsis relative *Thellungiella salsuginea* (*Eutrema salsugineum*). BMC Genomics 14:793 10.1186/1471-2164-14-793 24228715 PMC3832907

[B34] Lin Y.-F. Aarts M. G. M. (2012). The molecular mechanism of zinc and cadmium stress response in plants. Cell. Mol. Life Sci. 69, 3187–3206 10.1007/s00018-012-1089-z 22903262 PMC11114967

[B35] Lombi E. Zhao F. J. Dunham S. J. McGrath S. P. (2000). Cadmium accumulation in populations of *Thlaspi caerulescens* and *Thlaspi goesingense*. New Phytol. 145, 11–20 10.1046/j.1469-8137.2000.00560.x

[B36] McCarthy F. Wang N. Magee G. B. Nanduri B. Lawrence M. Camon E. (2006). AgBase: a functional genomics resource for agriculture. BMC Genomics 7:229 10.1186/1471-2164-7-229 16961921 PMC1618847

[B14] McGrath S. P. Sidoli C. M. D. Baker A. J. M. Reeves R. D. (1993). The potential for the use of metal-accumulating plants for the *in situ* decontamination of metal-polluted soils, in Integrated Soil and Sediment Research: A Basis for Proper Protection, (Proceedings of the European Conference on Integrated Research for Soil and Sediment Protection and Remediation, EUROSOL, MECC Maastricht, The Netherlands), eds Eijsackers H. J. P. Hamers T. (Dordrecht: Kluwer Academic Publishers), 673–676

[B37] Metzker M. L. (2010). Sequencing technologies - the next generation. Nat. Rev. Genet. 11, 31–46 10.1038/nrg2626 19997069

[B38] Meyer C.-L. Verbruggen N. (2012). The use of the model species *Arabidopsis halleri* towards phytoextraction of cadmium polluted soils. N. Biotechnol. 30, 9–14 10.1016/j.nbt.2012.07.009 22850245

[B39] Milner M. J. Kochian L. V. (2008). Investigating heavy-metal hyperaccumulation using *Thlaspi caerulescens* as a model system. Ann. Bot. 102, 3–13 10.1093/aob/mcn063 18440996 PMC2712422

[B40] Mohtadi A. Ghaderian S. Schat H. (2012). A comparison of lead accumulation and tolerance among heavy metal hyperaccumulating and non-hyperaccumulating metallophytes. Plant Soil 352, 267–276 10.1007/s11104-011-0994-5

[B41] Morozova O. Hirst M. Marra M. A. (2009). Applications of new sequencing technologies for transcriptome analysis. Annu. Rev. Genomics Hum. Genet. 10, 135–151 10.1146/annurev-genom-082908-145957 19715439

[B42] Niedringhaus T. P. Milanova D. Kerby M. B. Snyder M. P. Barron A. E. (2011). Landscape of next-generation sequencing technologies. Anal. Chem. 83, 4327–4341 10.1021/ac2010857 21612267 PMC3437308

[B43] Noret N. Meerts P. Vanhaelen M. Santos A. D. Escarré J. (2007). Do metal-rich plants deter herbivores? A field test of the defence hypothesis. Oecologia 152, 92–100 10.1007/s00442-006-0635-5 17216212

[B44] Nour-Eldin H. H. Andersen T. G. Burow M. Madsen S. R. Jorgensen M. E. Olsen C. E. (2012). NRT/PTR transporters are essential for translocation of glucosinolate defence compounds to seeds. Nature 488, 531–534 10.1038/nature11285 22864417

[B45] Ó Lochlainn S. Bowen H. C. Fray R. G. Hammond J. P. King G. J. White P. J. (2011). Tandem quadruplication of *HMA4* in the zinc (Zn) and cadmium (Cd) hyperaccumulator *Noccaea caerulescens*. PLoS ONE 6:e17814 10.1371/journal.pone.0017814 21423774 PMC3053397

[B46] Peer W. A. Mahmoudian M. Freeman J. L. Lahner B. Richards E. L. Reeves R. D. (2006). Assessment of plants from the *Brassicaceae* family as genetic models for the study of nickel and zinc hyperaccumulation. New Phytol. 172, 248–260 10.1111/j.1469-8137.2006.01820.x 16995913

[B47] Peer W. A. Mamoudian M. Lahner B. Reeves R. D. Murphy A. S. Salt D. E. (2003). Identifying model metal hyperaccumulating plants: germplasm analysis of 20 Brassicaceae accessions from a wide geographical area. New Phytol. 159, 421–430 10.1046/j.1469-8137.2003.00822.x 33873359

[B48] Pfalz M. Mikkelsen M. D. Bednarek P. Olsen C. E. Halkier B. A. Kroymann J. (2011). Metabolic engineering in *Nicotiana benthamiana* reveals key enzyme functions in Arabidopsis indole glucosinolate modification. Plant Cell 23, 716–729 10.1105/tpc.110.081711 21317374 PMC3077789

[B49] Plessl M. Rigola D. Hassinen V. Tervahauta A. Kärenlampi S. Schat H. (2010). Comparison of two ecotypes of the metal hyperaccumulator Thlaspi caerulescens (J. & C. PRESL) at the transcriptional level. Protoplasma 239, 81–93 10.1007/s00709-009-0085-0 19937357

[B50] Reeves R. D. Brooks R. R. (1983). European species of Thlaspi L. (Cruciferae) as indicators of nickel and zinc. J. Geochem. Explor. 18, 275–283

[B51] Remm M. Storm C. E. V. Sonnhammer E. L. L. (2001). Automatic clustering of orthologs and in-paralogs from pairwise species comparisons. J. Mol. Biol. 314, 1041–1052 10.1006/jmbi.2000.5197 11743721

[B52] Rigola D. Fiers M. Vurro E. Aarts M. G. M. (2006). The heavy metal hyperaccumulator *Thlaspi caerulescens* expresses many species-specific genes, as identified by comparative expressed sequence tag analysis. New Phytol. 170, 753–766 10.1111/j.1469-8137.2006.01714.x 16684236

[B53] Roosens N. H. Bernard C. Leplae R. Verbruggen N. (2004). Evidence for copper homeostasis function of metallothionein (MT3) in the hyperaccumulator *Thlaspi caerulescens*. FEBS Lett. 577, 9–16 10.1016/j.febslet.2004.08.084 15527754

[B54] Schweizer F. Fernández-Calvo P. Zander M. Diez-Diaz M. Fonseca S. Glauser G. (2013). Arabidopsis basic Helix-Loop-Helix transcription factors MYC2, MYC3, and MYC4 regulate glucosinolate biosynthesis, insect performance, and feeding behavior. Plant Cell 25, 3117–3132 10.1105/tpc.113.115139 23943862 PMC3784603

[B55] Slater G. Birney E. (2005). Automated generation of heuristics for biological sequence comparison. BMC Bioinformatics 6:31 10.1186/1471-2105-6-31 15713233 PMC553969

[B56] Sønderby I. E. Burow M. Rowe H. C. Kliebenstein D. J. Halkier B. A. (2010a). A complex interplay of three R2R3 MYB transcription factors determines the profile of aliphatic glucosinolates in Arabidopsis. Plant Physiol. 153, 348–363 10.1104/pp.109.149286 20348214 PMC2862430

[B57] Sønderby I. E. Geu-Flores F. Halkier B. A. (2010b). Biosynthesis of glucosinolates - gene discovery and beyond. Trends Plant Sci. 15, 283–290 10.1016/j.tplants.2010.02.005 20303821

[B58] Talke I. N. Hanikenne M. Kramer U. (2006). Zinc-dependent global transcriptional control, transcriptional deregulation, and higher gene copy number for genes in metal homeostasis of the hyperaccumulator *Arabidopsis halleri*. Plant Physiol. 142, 148–167 10.1104/pp.105.076232 16844841 PMC1557598

[B59] Ueno D. Milner M. J. Yamaji N. Yokosho K. Koyama E. Clemencia Zambrano M. (2011). Elevated expression of *TcHMA3* plays a key role in the extreme Cd tolerance in a Cd-hyperaccumulating ecotype of *Thlaspi caerulescens*. Plant J. 66, 852–862 10.1111/j.1365-313X.2011.04548.x 21457363

[B60] Van De Mortel J. E. Almar Villanueva L. Schat H. Kwekkeboom J. Coughlan S. Moerland P. D. (2006). Large expression differences in genes for iron and zinc homeostasis, stress response, and lignin biosynthesis distinguish roots of *Arabidopsis thaliana* and the related metal hyperaccumulator *Thlaspi caerulescens*. Plant Physiol. 142, 1127–1147 10.1104/pp.106.082073 16998091 PMC1630723

[B61] Van De Mortel J. E. Schat H. Moerland P. D. Ver Loren Van Themaat E. Van Der Ent S. Blankestijn H. (2008). Expression differences for genes involved in lignin, glutathione and sulphate metabolism in response to cadmium in *Arabidopsis thaliana* and the related Zn/Cd-hyperaccumulator *Thlaspi caerulescens*. Plant Cell Environ. 31, 301–324 10.1111/j.1365-3040.2007.01764.x 18088336

[B62] Verbruggen N. Juraniec M. Baliardini C. Meyer C.-L. (2013). Tolerance to cadmium in plants: the special case of hyperaccumulators. Biometals 26, 633–638 10.1007/s10534-013-9659-6 23881358

[B63] Wang H. Wu J. Sun S. Liu B. Cheng F. Sun R. (2011). Glucosinolate biosynthetic genes in Brassica rapa. Gene 487, 135–142 10.1016/j.gene.2011.07.021 21835231

[B64] Waterhouse A. M. Procter J. B. Martin D. M. A. Clamp M. Barton G. J. (2009). Jalview Version 2—a multiple sequence alignment editor and analysis workbench. Bioinformatics 25, 1189–1191 10.1093/bioinformatics/btp033 19151095 PMC2672624

[B65] Weber M. Harada E. Vess C. Roepenack-Lahaye E. V. Clemens S. (2004). Comparative microarray analysis of *Arabidopsis thaliana* and *Arabidopsis halleri* roots identifies nicotianamine synthase, a ZIP transporter and other genes as potential metal hyperaccumulation factors. Plant J. 37, 269–281 10.1046/j.1365-313X.2003.01960.x 14690510

[B66] Wu J. Zhao F.-J. Ghandilyan A. Logoteta B. Guzman M. Schat H. (2009). Identification and functional analysis of two ZIP metal transporters of the hyperaccumulator *Thlaspi caerulescens*. Plant Soil 325, 79–95 10.1007/s11104-009-0151-6

[B67] Yang R. Jarvis D. J. Chen H. Beilstein M. Grimwood J. Jenkins J. (2013). The reference genome of the halophytic plant Eutrema salsugineum. Front. Plant Sci. 4:46 10.3389/fpls.2013.00046 23518688 PMC3604812

