# Supplementary material for: A comprehensive set of transcript sequences of the heavy metal hyperaccumulator Noccaea caerulescens
Source: Front Plant Sci. 2014 Jun 20;5:261. doi: 10.3389/fpls.2014.00261 (PMC4064536; doi:10.3389/fpls.2014.00261)
Supplement: Supplementary file 2 [file DataSheet2.PDF]

SUPPLEMENTAL TABLE LEGENDS AND FIGURES

Table S1. *N. caerulescens* contigs and isotigs, with corresponding isogroups, with their BlastX best hit when compared to the Brassicaceae proteome dataset.
Only 16 contigs could not be assembled into an isotig and are listed at the top. The sequence length (in bp) as well as the number of reads constituting the contig or isotig is indicated.

Table S2. *N. caerulescens* isotigs with similarity to plant sequences found in the non-redundant NCBI GenBank protein database not represented in the Brassicaceae proteome dataset

Table S3. *N. caerulescens* isotigs with similarity to non-plant protein sequences.

Table S4. *N. caerulescens* isotigs with no similarity to any sequence in the NCBI nr database.

Table S5. Gene Ontology (GO) annotation of *N. caerulescens* isogroups

Table S6. GOSlim classification of annotated *N. caerulescens* GO terms.

Table S7. PlantCyc listing of biological pathways in which *N. caerulescens* isogroups are involved in.

Table S8. Genes involved in glucosinolate biosynthesis.

a

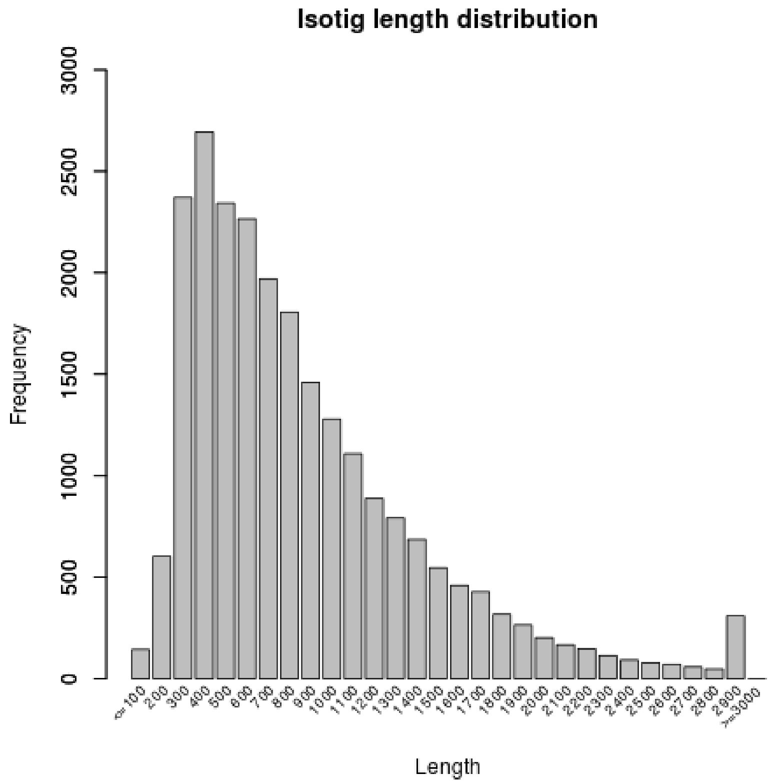

b

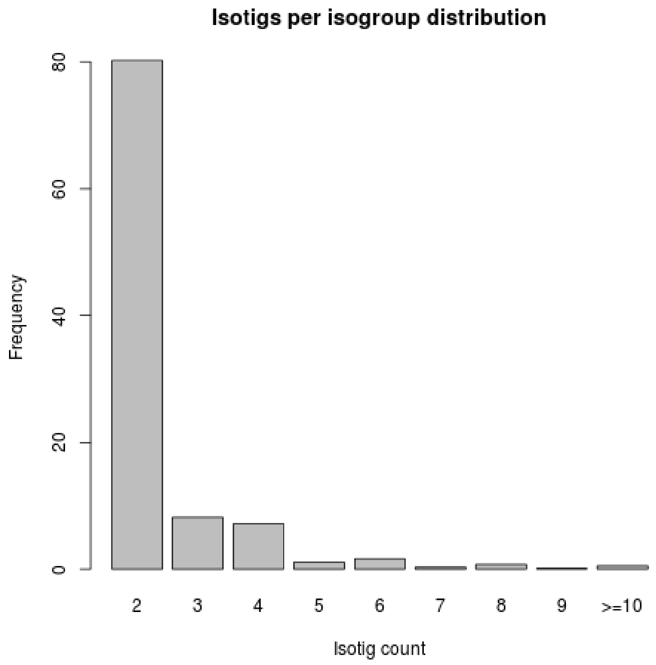

Fig S1. Analysis of N. caerulescens isotigs and isogroups

The frequency distribution of isotigs according to sequence length (a), the frequency distribution of the number of isotigs (isotig count) per isogroup (b) are shown.

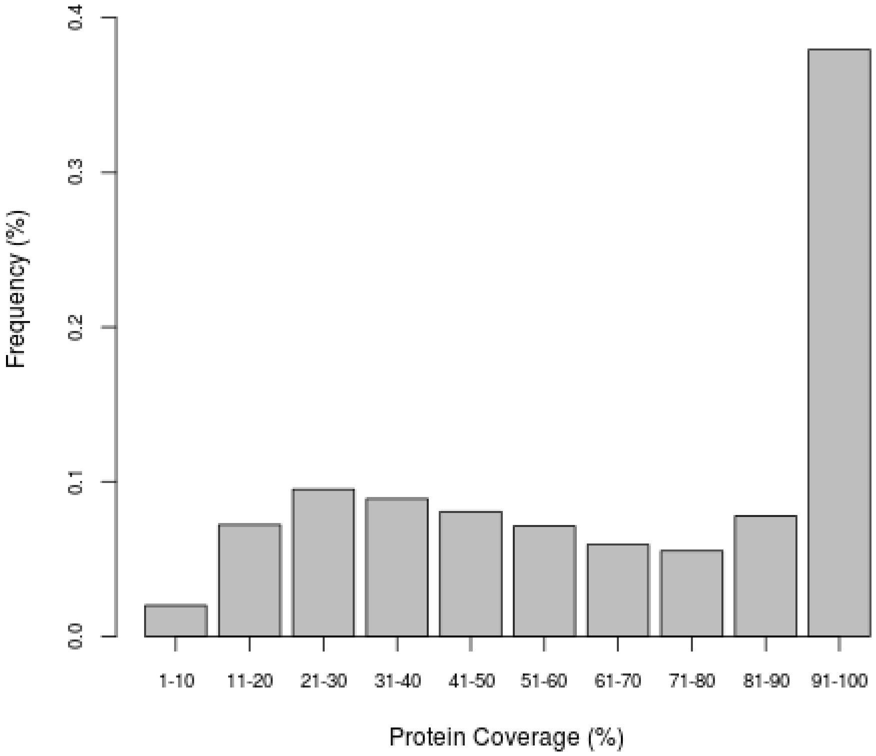

Figure S2. Frequency distribution of *N. caerulescens* isotigs according to percentage coverage of their Brassicaceae proteome best-hit.

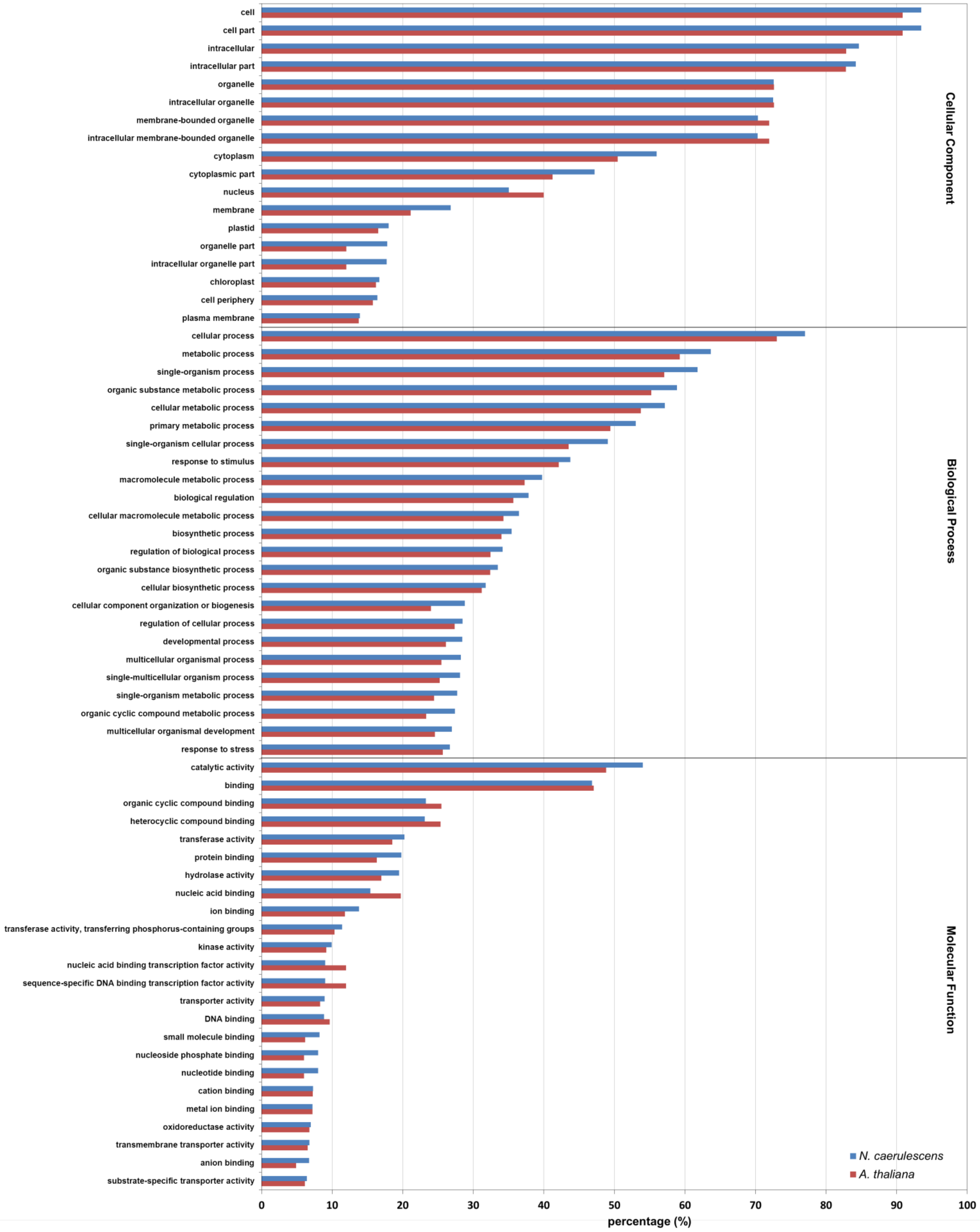

Fig S3. Comparative Gene Ontology (GO) classification of *N. caerulescens* and *A. thaliana*

N. caerulescens isogroups and A. thaliana genes were classified into three major functional groups: Cellular Component, Molecular Function, and Biological Process. Per group, the percentage of all isogroups or genes corresponding with the indicated GO-terms have been determined and the top 18 (Cellular Component) or top 24 (Biological Process and Molecular Function) of GO-terms are displayed.

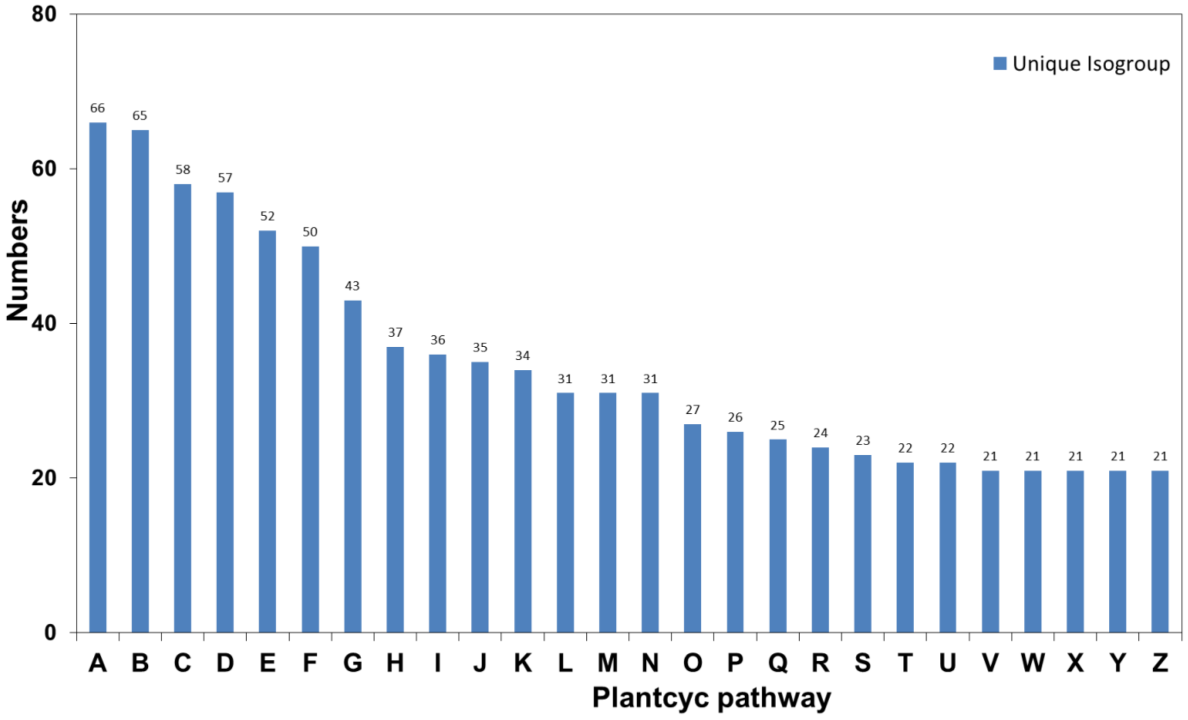

Fig S4. Main biological pathways in which Noccaea caerulescens genes are involved.

The number of N. caerulescens isogroups categorized in each of the top 26 pathways (A-X) are indicated upon comparison to the PlantCyc database. A, triacylglycerol degradation; B, homogalacturonan degradation; C, glycolysis II (from fructose-6P); D, tRNA charging; E, aerobic respiration (alternative oxidase pathway); F, betanidin degradation; G, photosynthesis light reactions; H, glutathione-mediated detoxification II; I, phospholipases; J, TCA cycle variation V (plant); K, sucrose degradation III; L, fatty acid &beta-oxidation II (peroxisome); M, coumarin biosynthesis (via 2-coumarate); N, phosphate acquisition; O, tetrahydrofolate biosynthesis II; P, lactose degradation III; Q, photorespiration; R, CDP-diacylglycerol biosynthesis II; S, 3-phosphoinositide biosynthesis; T, D-myo-inositol (1,4,5)-trisphosphate biosynthesis; U, callose biosynthesis; V, phenylalanine degradation III; W, xylan biosynthesis; X, adenosine nucleotides de novo biosynthesis; Y, starch biosynthesis; Z, trehalose biosynthesis I
